# The cytoplasmic domains of the CNNM family of transmembrane proteins modulate the ion channel-kinase TRPM7

**DOI:** 10.1016/j.jbc.2025.110720

**Published:** 2025-09-15

**Authors:** Sandra Tetteh, Pengyu Zong, Jianlin Feng, Emma L. Lee, Namariq Al-Saadi, Jeremy Willekens, Ayush Shah, Thushara Nethramangalath, Abigail L. Galeano, Haiyen Zheng, Kalle Gehring, Lixia Yue, Loren W. Runnels

**Affiliations:** 1 Department of Pharmacology, Rutgers-Robert Wood Johnson Medical School, Piscataway, New Jersey, USA; 2 Department Cell Biology, Calhoun Cardiology Center, UCONN Health Center, Farmington, Connecticut, USA; 3 Department of Biochemistry, McGill University, Montreal, Canada; 4 Biological Mass Spectrometry Resources, Robert Wood Johnson Medical School, Rutgers, State University of New Jersey, New Brunswick, New Jersey, USA; 5 Rutgers Cancer Institute, Rutgers, The State University of New Jersey, New Brunswick, New Jersey, USA

**Keywords:** TRPM7, CNNM, PTP4A, PRL, ARL15

## Abstract

The ion channel and kinase TRPM7 contribute to a wide range of physiological and pathological processes, yet how it is controlled by signaling pathways remains poorly understood. Members of the CNNM family of transmembrane proteins (CNNM1-4) have been shown to selectively bind and regulate the TRPM7 channel. CNNM-binding partners, including the PRL family of proteins (PRL1-3) and ARL15, also modulate TRPM7 channel function. However, the regulation of TRPM7's kinase activity *in vivo* remains unclear. CNNMs contain a DUF21 transmembrane domain, a CBS-pair domain, followed by a COOH-terminal CNBH domain. Here, we identified multiple interaction sites between TRPM7 and CNNMs. The CNNM transmembrane domain was sufficient to mediate assembly of the CNNM2-TRPM7 complex, while the CBS-pair and CNBH domains provided additional points of contact. Electrophysiological analysis revealed that the CBS-pair domain modulates TRPM7 channel activity. ARL15, a known suppressor of TRPM7 channel function, required the CNNM CBS-pair domain to inhibit channel activity. Additionally, the CNNM2 CNBH domain bound to the TRPM7 kinase domain and modestly enhanced its catalytic activity *in vitro*. Collectively, these findings demonstrate that the cytoplasmic domains of CNNMs play critical roles in regulating TRPM7 channel and kinase activities.

TRPM7, the first ion channel discovered to contain a kinase domain (1, 2), is permeable to divalent cations including Mg^2+^, Ca^2+^, and Zn^2+^ (3). Over the past 2 decades, ever-expanding physiological and pathological roles have been identified for the channel-kinase. In mice and *Xenopus laevis*, TRPM7 contributes to fertilization and early embryonic development (4, 5, 6, 7, 8, 9, 10). In mice, TRPM7 is also required for systemic Mg^2+^ homeostasis and intestinal Zn^2+^ absorption (9, 11). In zebrafish, TRPM7 influences melanophore survival—required for fish pigmentation—as well as sensory perception and renal handling of Mg^2+^ and Ca^2+^ (12, 13, 14, 15). In humans, mutations in TRPM7 are implicated in thrombopoiesis defects and Hallermann–Streiff syndrome, a congenital disorder that affects growth, cranial development, and the development of hair, eyes, and teeth (16, 17). Heterozygous mutations in TRPM7 have been identified in individuals with hypomagnesemia who simultaneously present with autism spectrum disorder and developmental delay (18). TRPM7 has been linked to several disease processes, including cancer cell metastasis (19, 20, 21), fibrosis (22, 23, 24), inflammation (25, 26, 27), neuronal ischemia (28, 29, 30), and most recently abdominal aortic aneurysms (31, 32). Remarkably, TRPM7 can influence extensive cellular processes *in vivo*, including apoptosis (33), receptor endocytosis (34), synaptic vesicle endocytosis (35), efferocytosis (36), insulin secretion (37), metabolism (38, 39, 40), cellular magnesium homeostasis (41), calcium influx (4, 20, 28, 42, 43, 44, 45, 46, 47), zinc transport (11, 48), chromatin remodeling (49), and cytoskeleton regulation (42, 50, 51, 52, 53). Despite the identification of key molecules that directly regulate the channel, including Mg^2+^, Mg·ATP, PIP_2_, halides, and protons, how this channel-kinase is controlled by signaling pathways in native systems remains poorly understood (1, 54, 55, 56, 57, 58).

Progress in understanding TRPM7's regulation *in vivo* accelerated with the identification of its binding partners-the cystathionine-β-synthase (CBS) pair domain divalent metal cation transport mediators (CNNMs: CNNM1, CNNM2, CNNM3, and CNNM4), also known as ancient conserved domain proteins (ACDPs) (59, 60). CNNMs transport Mg^2+^ and are regulated by the binding to ADP ribosylation factor-like GTPase 15 (ARL15) and members of the type IVA protein tyrosine (PTP4A) phosphatases, also known as phosphatases of regenerating liver (PRL1, PRL2, and PRL3) (61). CNNMs have been shown to stimulate Mg^2+^-efflux (62, 63)- a process that occurs independently of TRPM7 (59). However, when bound to TRPM7, CNNMs and their binding partners, ARL15 and PRLs, regulate TRPM7 channel function, mediating the influx of divalent cations into the cell (59, 60, 61, 64). Collectively, these data support a model in which CNNMs regulate Mg^2+^ homeostasis by controlling Mg^2+^efflux and influx in parallel (61). Nevertheless, the mechanisms by which CNNMs selectively regulate TRPM7—particularly through the actions of ARL15 and PRLs, which inhibit and stimulate TRPM7 channel function, respectively—remain poorly understood (59).

To gain insight into how CNNMs modulate TRPM7, we performed biochemical mapping experiments between TRPM7 and CNNM2, the isoform that mostly robustly interacts and stimulates TRPM7 function (59). We found that TRPM7 interacts with several distinct regions of CNNM2. The NH_2_-terminal transmembrane fragment of CNNM2 was sufficient for assembly with TRPM7. The CNNM CBS-pair domain interacted with both the cytosolic COOH- and NH_2_-terminal regions of TRPM7, whereas the CNBH domain exclusively interacted with the COOH-terminal region. Additionally, *in vitro* kinase assays revealed that the CNBH domain modulates TRPM7 kinase activity. TRPM7 phosphorylated the CNNM2 CNBH domain but not the CBS-pair domain, and multiple phosphorylation sites were identified on the CNBH domain by mass spectrometry. Furthermore, electrophysiology analysis demonstrated that the CBS-pair domain is vital for modulating the TRPM7 channel and is essential for ARL15-mediated suppression of channel function. Together, these findings demonstrate that CNNMs form multiple physical interactions with TRPM7 that are critical for regulating both its channel and kinase activities.

## Results

### CNNM2 NH_2_-terminus and transmembrane domain are sufficient for CNNM-TRPM7 assembly

**Figure 1:**
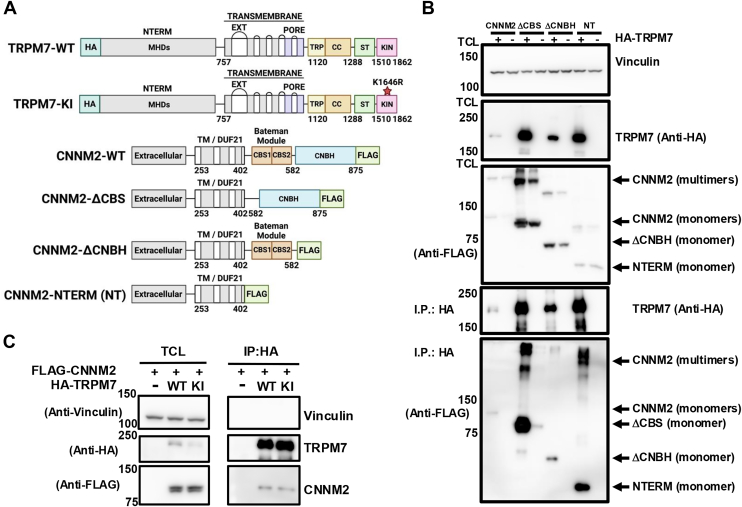
**CNNM2 NH_2_-terminal fragment is sufficient to interact with TRPM7.***A*, schematic representation of structural domains of TRPM7 and CNNM2 used in co-immunoprecipitation experiments. The cytoplasmic NH_2_-terminus (NTERM) of TRPM7 contains four Melastatin Homology Domains (MHD), followed by transmembrane domain segments that form the channel's pore. The cytoplasmic COOH-terminus includes a TRP box, a coiled-coil domain (CC), a serine/threonine (ST) rich region, and a serine/threonine-specific catalytic kinase domain (KIN). The NH_2_-terminus (NTERM) of CNNM2 contains an extracellular domain followed by a DUF21 transmembrane domain. The cytosolic COOH-terminus of CNNM2's includes a CBS-pair domain (composed of two cytosolic cystathionine-β-synthase (CBS)-pair motifs) that binds to Mg·ATP, PRL proteins, and ARL15 and a CNBH domain that is important for CNNM dimerization. *B*, HEK-293T cells were transfected with HA-tagged TRPM7 (HA-TRPM7) (+) or an empty vector control (−), along with the following FLAG-tagged CNNM2 constructs: full length CNNM2, CNNM2-ΔCBS (ΔCBS), CNNM2-ΔCNBH (ΔCNBH), or CNNM2-NTERM (NT). HA-TRPM7 was immunoprecipitated with magnetic HA-beads, and the immunopurified proteins were resolved by SDS-PAGE and analyzed by Western blotting. CNNM2 was detected as oligomers or monomers. as indicated. Cell culture media were supplemented with 20 mM MgCl_2_ to enhance CNNM and TRPM7 protein expression. *C*, HA-tagged TRPM7 (WT) or the kinase-inactive TRPM7-K1646R mutant (KI) was co-expressed in HEK-293T cells with FLAG-CNNM2. HA-TRPM7 was immunoprecipitated (I.P.) with magnetic HA-beads, and total cell lysates (TCL) and immunopurified proteins were analyzed by SDS-PAGE and Western blotting. See Figure S1 for uncropped images of the Western blots; and Figure S2 for the experimental replicate.

To gain insight into the mechanisms by which CNNMs modulate TRPM7 channel activity, we performed biochemical mapping experiments to identify the regions of interaction. Mammals have four CNNM isoforms (CNNM1-4), containing an N-terminal extracellular domain, a transmembrane domain (domain of unknown function 21 [DUF21]), and a large cytosolic region composed of a CBS-pair domain and a CNBH domain (Fig. 1*A*) (65). CBS-pair domains, also known as Bateman domains, consist of two repeated 60-residue CBS motifs that fold together to form a domain found in many eukaryotic and prokaryotic proteins (66, 67). CNNM CBS-pair domains dimerize in a head-to-head configuration, forming a disk-like structure that encloses a central nucleotide-binding site for Mg·ATP (68). The CNNM CBS-pair domain also binds to PRLs and ARL15, which regulate CNNM Mg^2+^-export activity (61, 62, 69, 70). Following the CBS-pair domain is the CNBH domain, which is critical for CNNM dimerization and function (71).

For our biochemical mapping experiments, we utilized CNNM2 fragments analogous to those employed by Hardy and colleagues to map the binding site of PRL2 on CNNM3. These included the CNNM2 NH_2_-terminus, which comprises the transmembrane domain (CNNM2-NT; residues 1–402); CNNM2 lacking the CNBH domain (CNNM2-ΔCNBH; residues 1–582); and CNNM2 lacking the CBS-pair domain (CNNM2-ΔCBS) (Fig. 1*A*) (69). Previous work demonstrated that overexpression of wild-type (WT) CNNM2 in HEK-293 decreases intracellular Mg^2+^ levels, which non-specifically impairs protein translation, including that of TRPM7 (59). Consistent with these findings, we observed that co-expression of WT CNNM2 with TRPM7 decreased channel expression compared to TRPM7 expressed alone in HEK-293 cells (Fig. 1*B*). This effect was reproducible across experiments (Fig. S2).

Deletion of either the CBS-pair domain or the CNBH domain from CNNM2 has been reported to disrupt Mg^2+^ efflux (71, 72). In agreement with these reports, we found that co-expression of CNNM2-ΔCBS or CNNM2-ΔCNBH with TRPM7 had a markedly reduced effect on channel expression compared to co-expression of wild-type CNNM2 with TRPM7. However, deletion of either the CBS-pair domain or the CNBH domain did not impair the interaction between CNNM2 and TRPM7 (Figs. 1*B* and S2). In particular, we found that NH_2_-terminus containing the transmembrane domain (CNNM2-NT) interacted with TRPM7 (Fig. 1*B*) and was sufficient to support the formation of the CNNM-TRPM7 complex.

The various CNNM2 constructs used in our study disrupt Mg^2+^ efflux to differing degrees, likely leading to variable intracellular Mg^2+^ levels in transfected cells and complicating efforts to quantify the interaction strength. Despite this, the interactions between these proteins were robust and reproducible (Fig. S2). We next assessed the specificity of the TRPM7-CNNM2 interaction and found that CNNM2 co-immunoprecipitated with TRPM7, whereas Mg^2+^ transporter SLC41A1-previously shown not to stimulate the TRPM7 channel, failed to interact with TRPM7 (Fig. S3) (59). CNNM2 also did not specifically interact with TRPM2 (Fig. S3), consistent with our previous studies indicating no interaction between TRPM2 and native CNNM3 or CNNM4 (59). It has been previously reported that deletion of the CBS-pair domain or CNBH domain does not interfere with the presentation of CNNM2 at the plasma membrane (71, 72). We observed similar results when we examined the localization of CNNM2-ΔCBS and CNNM2-ΔCNBH in cells compared to wild-type CNNM2 (Fig. S4*A*). Interestingly, simultaneous deletion of both domains disrupted CNNM2 cellular localization relative to wild-type CNNM2. When overexpressed in cells, CNNM2-NT localized to intracellular puncta where it co-localized with TRPM7 (Fig. S4*B*). These results suggest that the CBS and CNBH domains are both required for proper cellular localization of CNNM2, whereas the CNNM2-NT domain is sufficient for its association with TRPM7.

The role of TRPM7's kinase in channel activity has been extensively studied, but its influence on the channel's interaction with CNNMs remains unclear. Although it is widely accepted that the TRPM7 kinase does not participate in channel gating (41, 73, 74), mutations that abolish its catalytic activity have been shown to negatively affect TRPM7 protein stability (75). Specifically, these inactivating mutations enhance protein degradation *via* increased ubiquitination and promoted intracellular retention of the channel, underscoring a critical role for TRPM7 kinase activity in regulating TRPM7 trafficking and turnover (75).

To determine whether inactivation of the TRPM7 kinase affects its interaction with CNNM2, we transiently expressed CNNM2 with either TRPM7 or the TRPM7 kinase-inactive mutant, TRPM7-K1646R (TRPM7-KI), in HEK-293T cells (Fig. 1*C*). Similar to WT TRPM7, the TRPM7-KI mutant effectively and reproducibly co-immunoprecipitated CNNM2 (Figs. 1*C* and S2). These findings indicate that the kinase is not required for assembly of the CNNM2-TRPM7 complex.

**Figure 2:**
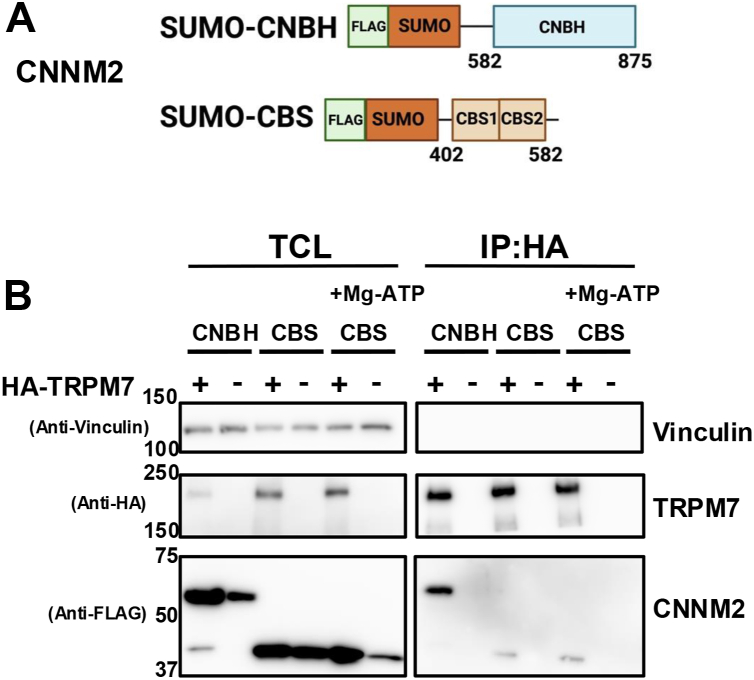
**TRPM7 interacts with CNNM2's cytosolic domains.***A*, schematic representation of structural domains of TRPM7 and CNNM2 used in co-immunoprecipitation experiments. The CNBH and CBS-pair domains, which are cytoplasmic facing in CNNM2, were fused with FLAG and Small Ubiquitin-like Modifier (SUMO) fusion tags to facilitate protein detection and expression, respectively. *B*, HA-tagged TRPM7 (HA-TRPM7) was co-transfected with FLAG-SUMMO-CNNM2-CNBH (SUMO-CNBH) or FLAG-SUMO-CNNM2-CBS (SUMO-CBS) in HEK-293T cells for 48 h. HA-TRPM7 was immunoprecipitated (I.P.), and both the total cell lysates (TCL) and immunopurified proteins were resolved by SDS-PAGE and analyzed by Western blotting using the indicated antibodies. Mg·ATP (1 mM) was included in the lysis buffer to assess its effect on the interaction of SUMO-CBS with TRPM7. See Figure S1 for uncropped blots and Figure S3 for the experimental replicate.

Our results indicate that the CNNM2 NH_2_-terminus, which contains the transmembrane domain, is sufficient for assembly of the CNNM2-TRPM7 complex. We next investigated whether additional interactions occur between their cytosolic regions. To enhance the expression and stability of CNBH and CBS-pair domains, we fused them to a SUMO tag (Fig. 2*A*). When SUMO-tagged CNBH (SUMO-CNBH) or CBS-pair (SUMO-CBS) domains were co-expressed with wild-type TRPM7, both SUMO-CNBH and SUMO-CBS co-immunoprecipitated with the channel (Fig. 2*B*); these results were reproducible (Fig. S5). The CBS-pair domain of CNNM family members, including CNNM2, binds to Mg·ATP, which can induce conformational changes (68). Our experiments revealed that the CBS-pair domain reproducibly interacted with wild-type TRPM7, even in the presence of 1 mM Mg·ATP in the lysis buffer (Figs. 2*B* and S5). These experiments demonstrated that, in addition to the interaction observed between TRPM7 and CNNM2's transmembrane domain, the cytosolic CNBH and CBS-pair domains also contribute to binding in a manner that is independent of-or at least not significantly altered by Mg·ATP.

### CNNM2 CBS-pair and CNBH domains interact with specific TRPM7 cytosolic regions

**Figure 3:**
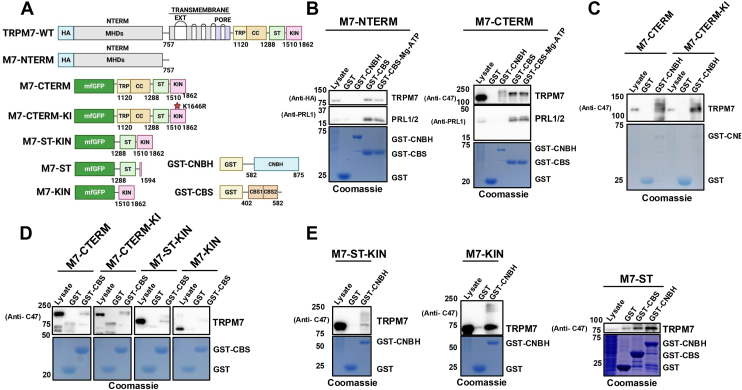
**CNNM2 cytosolic domains bind to specific regions on TRPM7 NH_2_- and COOH-termini.***A*, schematic representation of structural domains of TRPM7 and CNNM2 fragments used in the GST pull-down assays. *B*, glutathione agarose beads bound to bacterially expressed and purified GST, GST–CNBH, or GST-CBS were used to perform GST pull-down assays of the indicated TRPM7 NH_2_-terminal and COOH-terminal fragments fused to mfGFP in lysates from HEK-293 cells (see Experimental Procedures). Each TRPM7 fragment was expressed in HEK-293T cells for 48 h prior to lysis. Lysates were cleared by centrifugation, and the resultant supernatant incubated for 4 h with glutathione-agarose beads pre-bound with GST, GST–CNBH, or GST-CBS. Beads were then washed, and bound proteins were resolved by SDS-PAGE and analyzed by Western blotting using the indicated antibodies. *C*, GST pull-down assay using GST, GST–CNBH, and GST-CBS with M7-CTERM and kinase-inactive M7-CTERM-KI. *D*, GST pull-down assay using GST and GST–CBS with M7-ST-KIN and M7-KIN. *E*, GST pull-down assay using GST and GST–CNBH with M7-ST-KIN and M7-KIN. GST pull-down assay using GST, GST-CNBH, and GST-CBS with M7-ST. See Figure S1 for uncropped blots and Figure S4 for the experimental replicate.

To further elucidate the molecular interactions occurring between TRPM7 and CNNM2, we conducted GST pull-down purification assays using the CBS-pair domain and CNBH domains of CNNM2 (GST-CBS & GST-CNBH); to map their interaction with the channel's cytosolically exposed NH_2_- and COOH-terminal regions (Fig. 3*A*). The TRPM7 NH_2_-terminus is cytoplasmic-facing and contains four melastatin homology domains (MHDs), characteristic of TRPM family members. TRPM7 COOH-terminus is also cytoplasmic facing. Immediately following the pore domain is a highly conserved TRP box, a 25-amino acid motif conserved in almost all TRP channels, which contains the binding site for the TRPM7 activator naltribren, underscoring the importance of the TRP box in channel gating (76). Located after the TRP box is a cytoplasmic coiled-coil domain (1), which has been reported to influence TRPM7 sensitivity to Mg nucleotides (77). A serine/threonine (ST)-rich region located after the coiled-coil domain is extensively autophosphorylated by TRPM7's kinase and facilitates interactions with substrates, enhancing their phosphorylation (78). The catalytic kinase domain resides at TRPM7's COOH-terminus.

In GST pull-down assays, the CNNM2's CBS-pair domain consistently interacted with both the NH_2_- and COOH-terminal region of TRPM7 (M7-CTERM and M7-NTERM, respectively). In contrast, the CNNM2 CNBH domain exclusively and reproducibly interacted with the COOH-terminal region (Figs. 3*B* and S6). Given that the CNNM2 CBS-pair domain binds to Mg·ATP, we tested whether Mg·ATP would interfere with its binding to the TRPM7 COOH terminal region. Inclusion of 1 mM Mg·ATP into the lysate did not disrupt binding (Figs. 3*B* and S6). As a positive control for our assays, we probed for PRL1/2, which is known to interact with CNNM2's CBS-pair domain (79, 80). As expected, we detected an interaction between PRLs and the CBS-pair domain of CNNM2 and confirmed this interaction under our assay conditions (Figs. 3*B* and S6). We next investigated the interaction of the CNBH domain with TRPM7 COOH-terminus.

A GST pull-down assay using GST-CNBH reproducibly yielded a more diffuse band of the TRPM7 COOH-terminal domain compared to that observed with GST-CBS, which contains the CBS-pair domain (Figs. 3*B* and S6). One possibility is that GST-CNBH is also co-purified endogenous TRPM7 COOH-terminal fragments (referred to as M7CKs) that are expressed in HEK-293 cells (49). Another possibility is that the CNBH domain alters TRPM7 autophosphorylation. TRPM7's COOH-terminal domain contains a coiled-coil domain, followed by the serine/threonine (ST) rich region that undergoes significant autophosphorylation by the channel's kinase (78, 81). To test whether autophosphorylation accounts for the diffuse banding pattern, we conducted GST pull-down assays using GST-CNBH and a kinase-inactive TRPM7 COOH-terminal domain (M7-CTERM-KI). Both wild-type and kinase-inactive TRPM7 COOH-terminal domains reproducibly interacted with the GST-CNBH domain and displayed similar banding patterns (Figs. 3*C* and S6), indicating that the diffuse bands were not due to autophosphorylation. GST-CBS also reproducibly interacted with both the WT (M7-CTERM) and kinase inactive (M7-CTERM-KI) TRPM7 COOH-terminal domains (Figs. 3*D* and S6).

To determine which regions of the TRPM7 COOH-terminal region interact with the CBS-pair and CNBH domains, we performed GST pull-down assays using the serine-threonine (ST) rich region with the catalytic kinase domain of TRPM7 (M7-ST-KIN; 1288–1862), the catalytic kinase domain alone (M7-KIN; 1510–1862), as well the ST rich region alone (M7-ST; 1288–1594) (Fig. 3*A*). The GST-CBS domain of CNNM2 reproducibly interacted with the M7-CTERM fragment (1120–1862), the M7-ST-KIN fragment (1288–1862) but not with the isolated kinase domain (1510–1862) (Figs. 3*D* and S6). These results indicate that the CBS domain does not bind the TRPM7 kinase domain itself but likely interacts with TRPM7's ST rich region. To confirm this, we conducted a pull-down assay with the ST rich region of TRPM7 (M7-ST; 1288–1594) and detected an interaction with the GST-CBS domain (Figs. 3*E* and S6). Taken together, our results show that the CBS-pair domain of CNNM2 interacts with TRPM7's ST rich region in the COOH-terminal domain as well as with TRPM7's NH_2_-terminal domain. To further map the interaction sites of the CNBH domain, we performed pull-down assays using the GST-CNBH and lysates from HEK-293 cells expressing M7-ST-KIN (1288–1862), M7-KIN (1510–1862), and M7-ST (1288–1594) (Fig. 3*A*). The CNBH domain interacted with all three fragments, including the isolated catalytic kinase domain (M7-KIN; 1510–1862) (Figs. 3*E* and S6). Interactions were also detected between GST-CBS and the ST rich region of TRPM7 (Figs. 3*E* and S6), indicating that both the CNBH and CBS-pair domains engage with the ST rich region. Notably, only the CNBH domain exhibited binding to TRPM7's kinase domain.

### CNNM2's CNBH modulates TRPM7 kinase activity and is phosphorylated *in vitro*

**Figure 4:**
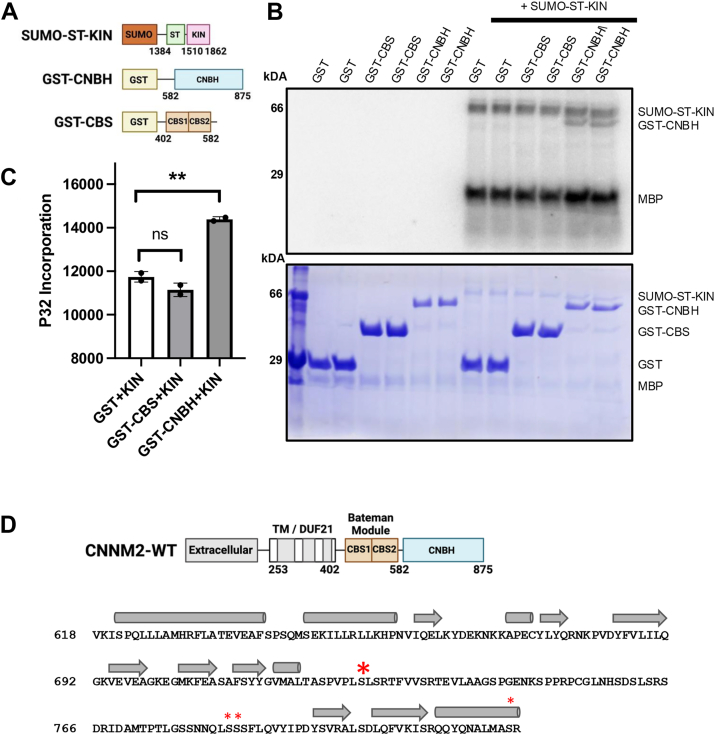
**CNNM2's CNBH domain modestly stimulates TRPM7 kinase activity.***A*, schematics of protein constructs used for the *in vitro* kinase assay. *B*, a bacterially purified SUMO-tagged TRPM7 fragment containing portions of the ST rich region and the catalytic kinase domain (SUMO-ST-KIN) was used in an *in vitro* kinase assay using myelin basic protein (MBP) as a generic substrate. GST-tagged CNBH (GST-CNBH) and CBS-pair domain (GST-CBS) were included in the assay to assess their effects on TRPM7 kinase activity. GST served as a negative control. The autoradiograph shows incorporation of ^32^P into MBP, SUMO-M7-ST-KIN, and GST-CNBH. Phosphorylation of GST-CNBH but not GST-CBS and GST indicate that the CNBH domain is a substrate of the TRPM7 kinase. The corresponding Coomassie-stained SDS-PAGE gel shows the protein inputs for the 6 min kinase reaction. Individual biological replicates are shown. *C*, quantification of the ^32^P incorporation into MBP from panel (*B*). GST-CBS did not significantly (*p* = 0.1644, unpaired two-tail *t* test) alter SUMO-ST-KIN kinase activity compared controls (GST and MBP alone). In contrast, GST-CNBH modestly stimulates the kinase activity of SUMO-ST-KIN (*p* = 0.0052, unpaired two-tail *t* test), and this effect was reproducible (Fig. S6). *D*, protein sequence of mouse CNNM2 CNBH domain. Secondary structure elements are based on the published structure of the human CNNM2 CNBH domain (71), with alpha helices shown as a tube and beta sheets as arrows). The purified GST-CNBH domain was phosphorylated by SUM-ST-KIN to saturation through overnight incubation. On-line digestion and subsequent LC-MS/MS spectrometry (see Experimental Procedures) identified multiple *in vitro* phosphorylation sites by TRPM7 kinase, indicated by the *red asterisks*. The most prominent site was Ser726 (*large red asterisk*); additional sites included Ser783, Ser784, and Ser819. The statistical analysis was performed using GraphPad Prism version 10.0.1 (218) and Student's *t* test (two-tailed) ∗∗*p* = 0.0052. “ns” indicates “not significant.”

It was previously reported that co-expression of CNNM3 with TRPM7 in HEK-293 cells suppressed the autophosphorylation of TRPM7 at Ser1511, suggesting that CNNM3 affects TRPM7 kinase activity (60). Our co-immunoprecipitation and GST pull-down assays demonstrated that both the GST-CBS and GST-CNBH domains of CNNM2 interact with the serine/threonine (ST)-rich region. However, GST-CNBH additionally binds to TRPM7's kinase domain. The impact of CNNM2 on TRPM7 kinase activity has not been reported. We therefore investigated the impact of purified GST-CBS and GST-CNBH domains on TRPM7 kinase catalytic activity. For this experiment, we utilized a bacterially purified SUMO-tagged kinase domain that contains a portion of the ST rich region SUMO-ST-KIN (1384–1862), which was previously employed to study TRPM7 autophosphorylation (81). *In vitro* kinase assays revealed that the GST-CBS domain did not stimulate SUMO-ST-KIN-mediated phosphorylation of myelin basic protein (MBP), a generic kinase substrate, compared to samples with GST as a negative control (Fig. 4). Interestingly, inclusion of GST-CNBH domain with SUMO-ST-KIN modestly stimulated TRPM7-kinase activity compared to GST alone (Fig. 4, *B* and *C*). The stimulatory effect of GST-CNBH on TRPM7 kinase activity was reproducible (Fig. S7). Surprisingly, our experiments revealed phosphorylation of CNNM2's CNBH domain but not its CBS domain by the SUMO-ST-KIN (Fig. 4*B*). Using online pepsin digestion and followed by LC-MSMS spectrometry (see Experimental Procedures), we identified several *in vitro* TRPM7 kinase phosphorylation sites on the CNBH domain (Table S2, S726: 71.5% phosphorylation, S783/S784: 2.2%, S819: 1.3%). Thus, although CNNM2 CBS-pair domain does not directly alter the catalytic activity of the TRPM7 kinase, the CNBH domain both stimulates kinase activity and serves as a substrate. However, the effects of these domains on TRPM7 channel activity remain unexplored.

### CNNM CBS-pair domain modulates TRPM7 channel activity

**Figure 5:**
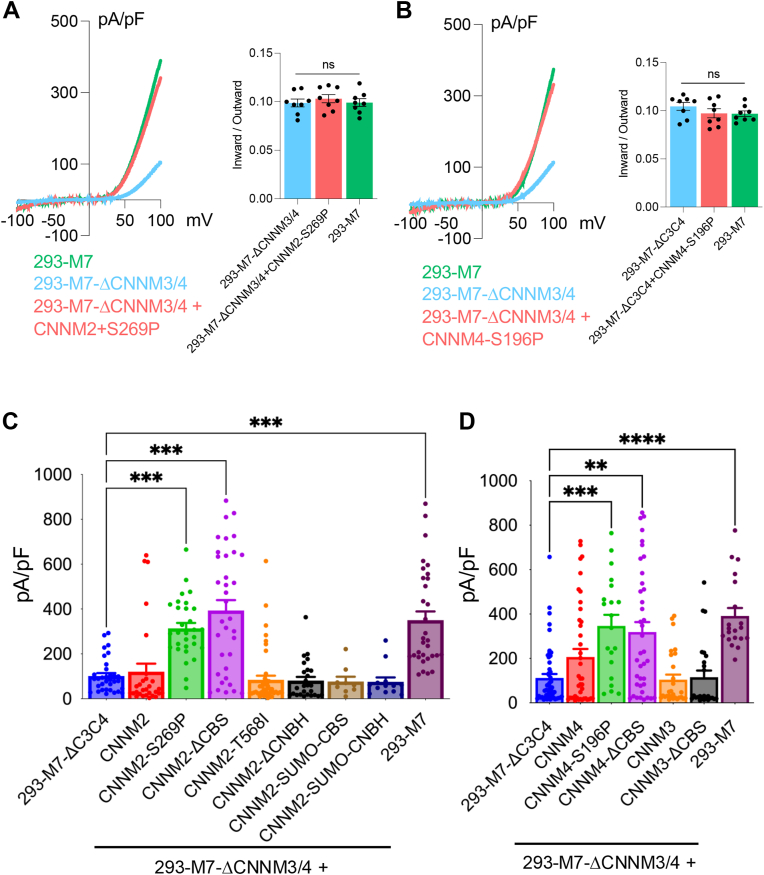
**Electrophysiological analysis of the effect of CNNMs on TRPM7 channel activity.** Whole-cell electrophysiological recordings of TRPM7 transiently overexpressed in 293-M7-ΔCNNM3/4 cells, which are deficient in CNNM3 and CNNM4 protein expression and express FLAG-tagged TRPM7 in response to tetracycline. An internal pipette solution lacking Mg^2+^ and Mg·ATP was used to elicit full TRPM7 channel activity. *A*, co-expression of CNNM2-S269P in 293-M7-ΔCNNM3/4 cells increased TRPM7 current densities to levels comparable to those in wild-type 293-M7 cells. The inward to outward current density ratio at −100 mV and + 100 mV, respectively, was similar across all three conditions. Individual biological replicates are shown. One way ANOVA (F = 0.3585, *p* = 0.7029). *B*, co-expression of CNNM4-S196P in 293-M7-ΔCNNM3/4 cells also elevated TRPM7 current densities to levels seen in wild-type 293-M7 cells. The ratio of inward to outward current densities at −100 mV and + 100 mV, respectively, remained consistent among the groups. One way ANOVA (F = 1.188, *p* = 0.3244). *C*, effect of co-expression of the indicated CNNM2 variants on TRPM7 outward current densities measured at +100 mV in 293-M7-ΔCNNM3/4 cells. Individual biological replicates are shown. Normality within each group was assessed with the Shapiro–Wilk test (GraphPad Prism v10.0.1). Variance homogeneity was evaluated with the Brown–Forsythe test on log_10_-transformed values. Because several groups were non-normal and variances were unequal, we used a Kruskal–Wallis test, which showed a significant overall effect, H(8) = 109.6, *p* < 0.0001, N = 252 (ε^2^ = 0.418). Pre-specified *post hoc* pairwise comparisons *versus* 293-M7ΔC3C4 were performed using Dunn's test with Holm correction (two-sided α = 0.05). Significance: *p* ≤ 0.01 (∗), *p* ≤ 0.001 (∗∗*), p ≤ 0.0001 (*∗∗∗). 293-M7ΔC3C4 *versus* CNNM2-S269P (*p* = 0.002). 293-M7ΔC3C4 *versus* CNNM2-ΔCBS (*p* = 0.004). 293-M7ΔC3C4 *versus* 293-M7 (*p* = 0.002). Non-significant comparisons are not shown. *D*, effect of co-expression of indicated CNNM4 and CNNM3 variants on TRPM7 current densities measured at +100 mV in 293-M7-ΔCNNM3/4 cells. Individual biological replicates are shown. A Kruskal–Wallis test was again used as the primary analysis. Normality within each group was assessed with the Shapiro–Wilk test (GraphPad Prism v10.0.1). Variance homogeneity was evaluated with the Brown–Forsythe test on log_10_-transformed values. Because several groups were non-normal and variances were unequal, we used a Kruskal–Wallis test, which showed a significant overall effect, H(6) = 52.46, *p* = 1.5 × 10^-9^, N = 225 (ε^2^ = 0.213). Pre-specified *post hoc* pairwise comparisons *versus* 293-M7ΔC3C4 were performed using Dunn's test with Holm correction (two-sided α = 0.05). 293-M7ΔC3C4 *versus* CNNM4-S196P (*p* = 0.0004), 293-M7-ΔC3C4 *versus* CNNM4-ΔCBS (*p* = 0.0029), and 293-M7-ΔC3C4 *versus* 293-M7 (*p* = 0.0001). Significance: *p* ≤ 0.01 (∗), *p* ≤ 0.001 (∗∗*), p ≤ 0.0001 (*∗∗∗), *p ≤ 0.00001 (*∗∗∗∗). Non-significant comparisons are not shown.

In our previous investigation of the effect of CNNMs on TRPM7, co-expression of CNNMs with the channel in HEK-293 cells stably expressing TRPM7 (293-M7 cells) significantly increased channel function (59). In contrast, knockout of CNNM3 and CNNM4 in WT HEK-293 cells (293-ΔCNNM3/4 cells) or in HEK-293 cells stably expressing FLAG-tagged mTRPM7 (293-M7-ΔCNNM3/4) substantially reduced TRPM7 current amplitudes compared to the WT 293-M7 control line (59). Importantly, knockout of CNNM3 and CNNM4 did not affect the surface expression levels of TRPM7, indicating that CNNMs potentially affect TRPM7 through another mechanism (59). Restoration of TRPM7 channel function in 293-M7-ΔCNNM3/4 cells was achieved using stable episomal expression of CNNM4, which kept CNNM4 protein levels sufficiently low (59). This approach was necessary because, as mentioned above, transient overexpression of CNNM2 or CNNM4 in HEK-293 cells decreased cytosolic Mg^2+^ levels, which we found nonspecifically interfered with protein translation, including that of TRPM7 (59). To facilitate electrophysiological analysis of the effect of CNNMs on TRPM7 channel function by transient transfection, we took advantage of our discovery that the introduction of the S196P mutation, which is associated with Jalili syndrome (82), at the putative CNNM Mg^2+^ binding site found in the transmembrane domain of CNNM4 (83)—completely disrupted CNNM4's capacity to lower intracellular Mg^2+^ (59). Importantly, the S196P mutation does not interfere with CNNM4's ability to stimulate TRPM7-dependent divalent cation influx (59). TRPM7-mediated divalent influx by the CNNM4-S196P mutant was significantly higher than that produced by WT CNNM4 (59). We, therefore, investigated whether the introduction of the analogous mutation in CNNM2 (S269P) could be similarly exploited to allow transient transfection experiments in our electrophysiological analysis. Indeed, we found that compared to WT CNNM2, transient transfection of CNNM2-S269P, as well as the equivalent mutation in CNNM4 (CNNM4-S196P), effectively restored TRPM7 channel activity in 293-M7-ΔCNNM3/4 cells (Fig. 5, *A* and *B*). It is worth noting that we did not observe any evidence that CNNMs are affecting the TRPM7 current-voltage relationship. The recovered current in 293-M7-ΔCNNM3/4 + CNNM2-S269P or 293-M7-ΔCNNM3/4 + CNNM4-S196P had the same ratio of current densities (inward *versus* outward) as measured in 293-M7 cells (Fig. 5, *A* and *B*). These data indicate that CNNMs are not affecting TRPM7's voltage dependence, as the ratio of inward to outward currents remains unchanged. Although CNNM2-S269P disables Mg^2+^-efflux activity, it appears that CNNMs can stimulate the TRPM7 channel independently of CNNMs Mg^2+^-export activity. Since we previously found that knockout of CNNM3 and CNNM4 does not affect the surface expression levels of TRPM7 (59), we speculate that CNNMs affect TRPM7 by influencing the gating or by increasing the number of functional channels.

Our biochemical data indicate that the cytoplasmic domains of CNNMs interact with TRPM7. Previously, we demonstrated that re-expression of CNNM4 restored TRPM7 channel activity in 293-M7-ΔCNNM3/4 cells by reexpression of CNNM4 (Fig. 5*D*) (59). To better understand how the cytoplasmic domains of CNNMs contribute to the regulation of TRPM7, we employed a gain-of-function approach to assess the effects of deleting CNNM2's CBS-pair domain or its CNBH domains. The absence of these domains have been previously been shown to also disable CNNM-mediated Mg^2+^-export activity (71, 72), Deletion of the CNNM2 CNBH domain prevented the restoration of TRPM7 channel activity in 293-M7-ΔCNNM3/4 cells (Fig. 5*C*), whereas deletion of the CNNM2 CBS-pair domain did not, as CNNM2-ΔCBS co-expression with TRPM7 successfully rescued channel function (Fig. 5*C*). Furthermore, co-expression of the individual CBS-pair or CNBH domains with TRPM7 failed to enhance current amplitude compared to non-transfected cells (Fig. 5*C*), suggesting that CNNM2's NH_2_-terminal and transmembrane domain are required for CNNM2 to restore TRPM7 channel function.

Next, we examined deletion of the CBS-pair domain in the other CNNM isoforms. The CBS-pair domain deletion in CNNM4, which is known to disrupt Mg^2+^efflux activity (72), did not impair TRPM7 currents restored in 293-M7-ΔCNNM3/4 cells when CNNM4-ΔCBS and TRPM7 were co-expressed (Fig. 5*D*). Strikingly, neither co-expression of CNNM3 nor of CNNM3 lacking the CBS-pair domain (CNNM3-ΔCBS) with TRPM7 in 293-M7-ΔCNNM3/4 cells increased TRPM7 current amplitudes compared to cells expressing TRPM7 alone (Fig. 5*D*). This result is consistent with the electrophysiological findings of Kollewe and colleagues, who similarly found that overexpression of CNNM3 had little impact on TRPM7 current amplitudes when co-expressed with the channel in HEK-293 cells (60). Thus, CNNM2 and CNNM4 appear to be the isoforms most capable of stimulating TRPM7 channel activity when these proteins are heterologously co-expressed.

Our data thus far point to a role for the CBS-pair domain in regulation of TRPM7 channel activity. Binding of Mg·ATP to the CBS-pair domain has been shown to trigger a conformational change from a twisted structure to a flat disk-like conformation that primarily affects the structural elements connecting the CBS-pair domain with the transmembrane region (68, 84). The T568I mutation located within the CBS-pair domain is associated with dominant hypomagnesemia (85). Like nucleotide binding to the CBS-pair domain, the T568I amino acid substitution also promotes a conformational change in the CBS-pair domain from a twisted structure to a flat disc-like conformation (68). The T568I substitution in the CBS-pair domain of CNNM2 (CNNM2-T568I) impaired its ability to restore TRPM7 channel activity in 293-M7-ΔCNNM3/4 cells when co-expressed with TRPM7 (Fig. 5*C*).

**Figure 6:**
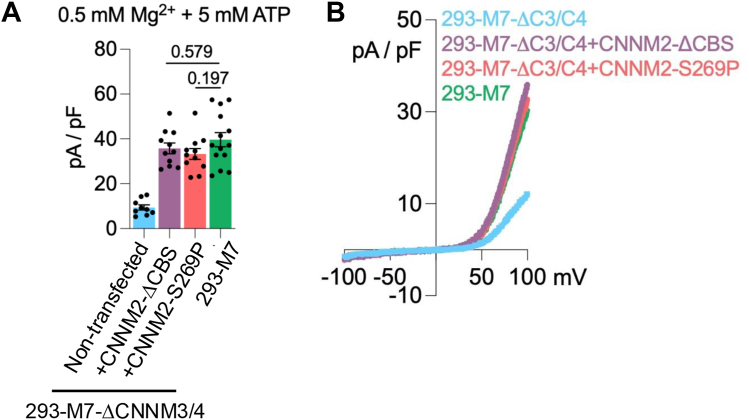
**Mg·ATP does not prevent CNNM2-S269P from stimulating the TRPM7 channel.***A*, whole-cell patch-clamp recordings of TRPM7 transiently overexpressed in 293-M7-ΔCNNM3/4 cells, which lack CNNM3 and CNNM4 protein expression and express FLAG-tagged TRPM7 in response to tetracycline. An internal pipette solution containing Mg^2+^ and Mg·ATP was used to suppress maximal TRPM7 channel activity. Co-expression of CNNM2-S269P mutant or CNNM2-ΔCBS mutant with TRPM7 similarly increased TRPM7 channel activity in 293-M7-ΔCNNM3/4 cells to levels comparable to 293-M7 cells expressing TPRM7 (+ tetracycline). Individual biological replicates are shown. *B*, representative TRPM7 whole-cell current traces are shown. The statistical analysis was performed using GraphPad Prism version 10.0.1 (218). A Kruskal–Wallis test was employed, which showed a significant overall effect, H(3) = 22.41, *p* = 5.36 × 10^-5^, N = 45 (ε^2^ = 0.473). Pre-specified *post hoc* pairwise comparisons *versus* 293-M7 were performed using Dunn's test with Holm correction (two-sided α = 0.05). 293-M7-ΔCNNM3/4 was significantly lower than 293-M7 (*p* = 2.22 × 10^-5^). The other calculated *p* values are shown, which were not significant.

As TRPM7 is regulated by Mg·ATP (54), we examined whether the CBS-pair domain of CNNM2 modulates TRPM7 channel function through Mg·ATP. Inclusion of Mg·ATP in the internal pipette solution reduced TRPM7 current densities in wild-type HEK-293 cells expressing TRPM7, in 293-M7-ΔCNNM3/4 cells expressing CNNM2-S269P, or in 293-M7-ΔCNNM3/4 cells expressing CNNM2-ΔCBS to similar levels, with no significance differences detected (Fig. 6, *A* and *B*). Thus, the CBS-pair domain appears not to be involved in Mg·ATP regulation of the TRPM7 channel under the conditions tested. Given that the CBS-pair domain of CNNMs binds to PRLs and ARL15 (62, 69, 70), it remains possible that this domain serves as an avenue by which these proteins regulate TRPM7.

### CNNM2 CBS-pair domain is required for ARL15-dependent regulation of TRPM7

The CBS-pair domain of CNNMs has been shown to bind to PRLs through a loop that extends into the phosphatase active site of PRLs (79, 80, 86). Recently, the structure of the CBS-pair domain in complex with ARL15 was determined (61). ARL15 competes with PRLs for binding to the CBS-pair domain (61, 64), and both ARL15 and PRLs have been shown to negatively regulate CNNM Mg^2+^-efflux (61, 62). CNNMs, PRLs, and ARL15 have also been shown to regulate TRPM7, pointing to a novel mechanism for the channel's *in vivo* control (59, 60, 61, 64). Nevertheless, how CNNMs influence TRPM7 function remains poorly understood.

Among the proteins that bind to the CBS-pair domain, the strongest evidence that they modulate TRPM7 channel function comes from studies of the ARL15 protein. ARL15 is a member of the RAS superfamily of small GTPases that regulates glycosylation and Mg^2+^-transport activity of CNNM2 (70). ARL15 was also found to associate with native TRPM7 and CNNMs in high molecular weight complexes isolated from brain (60). Overexpression of ARL15 reduced TRPM7 current amplitudes of the overexpressed channel and decreased TRPM7 channel activity in a zinc influx assay (59, 60). ARL15 competes with PRLs to bind CNNM2 through its CBS-pair domain (61, 70). Recently, the structure of ARL15 in complex with the CBS-pair domain was solved, enabling the identification of amino acid residues that when mutated disrupt the binding between ARL15 and the CNNM2 CBS-pair domain (61). The mutation of R95A in human ARL15 (ARL15-R95A) was found to disrupt the binding of ARL15 to the CBS-pair domain. Whereas co-expression of WT ARL15 with TRPM7 suppressed TRPM7 channel activity, co-expression of the ARL15-R95A mutant did not (61).

**Figure 7:**
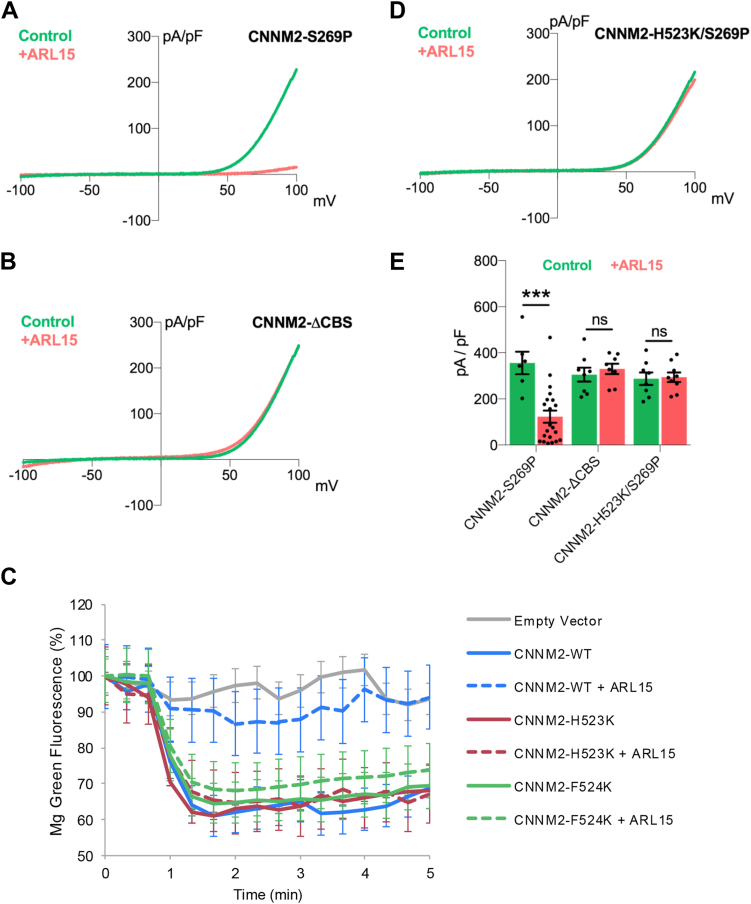
**Disruption of CNNM-binding site for ARL15 blocks regulation of CNNM-dependent cation transport.***A*, whole-cell electrophysiological recordings of TRPM7 transiently overexpressed in 293-M7-ΔCNNM3/4 cells, which lack CNNM3 and CNNM4 protein expression and express FLAG-tagged TRPM7 in response to tetracycline. An internal pipette solution lacking Mg^2+^ and Mg·ATP was used to achieve full TRPM7 channel activity. Co-expression of CNNM2-S269P mutant with TRPM7 increased TRPM7 channel activity. Transient co-expression of the CNNM-binding protein ARL15 with CNNM2-S269P and TRPM7 suppressed TRPM7 channel activity. A representative TRPM7 whole-cell current trace is shown. *B*, co-expression of CNNM2-ΔCBS mutant with TRPM7 also increased TRPM7 channel activity in 293-M7-ΔCNNM3/4 cells. However, transient co-expression of the CNNM-binding protein ARL15 with CNNM2-ΔCBS and TRPM7 failed to suppress TRPM7 channel activity. A representative TRPM7 whole-cell current traces is shown. *C*, ARL15 inhibited Mg^2+^ efflux by WT CNNM2 but not the H523K and F524K mutants. HEK-293T cells transfected with the indicated constructs were loaded with a magnesium sensitive dye and fluorescence was monitored following Mg^2+^ depletion. The mean relative fluorescence intensity of 10 cells is plotted. *D*, co-expression of CNNM2-H523K/S269P mutant with TRPM7 increased TRPM7 channel activity in 293-M7-ΔCNNM3/4 cells. Transient co-expression of the CNNM-binding protein ARL15 with CNNM2-H523K/S269P and TRPM7 did not suppress TRPM7 channel activity. Representative TRPM7 whole-cell current traces are shown. *E,* Graphic representation of outward TRPM7 current densities at + 100 mV obtained from conditions described above in (*A*), (*B*), and (*D*). Individual biological replicates are shown. Group means were compared with unpaired, two-sided *t*-tests (GraphPad Prism); false discovery rate (q) was controlled across tests (Benjamini–Hochberg). CNNM2-S269P showed a marked reduction with +ARL15 (356.1 vs 122.8; mean difference 233.2 ± 55.8 SE; t (25) = 4.178, *p* = 3.13 × 10^−4^, q = 6.32 × 10^−4^; 95% CI [118, 348]), whereas CNNM2-ΔCBS (305.4 vs 330.4; t (14) = 0.669, *p* = 0.514, q = 0.519; 95% CI [−105, 55]) and CNNM2-H523K/S269P (287.6 vs 294.1; t (15) = 0.193, *p* = 0.850, q = 0.572; 95% CI [−78, 65]) were not different. Thus, after multiple-testing correction, only CNNM2-S269P was significant. ∗∗∗*p* ≤ 0.001. N.S. indicates “not significant”.

To further elucidate the role of the CNNM2 CBS-pair domain in TRPM7 channel regulation, we examined the effect of overexpression of wild-type ARL15 on TRPM7 channel activity when co-expressed with CNNM2-S269P and TRPM7 in 293-M7-ΔCNNM3/4 cells. We observed that wild-type ARL15 significantly reduced TRPM7 channel activity, consistent with previous reports (Fig. 7, *A*–*E*) (60, 61). In contrast, co-expression of WT ARL15 with CNNM2-ΔCBS and TRPM7 did not reduce TRPM7 channel currents (Fig. 7, *B*–*E*), underscoring the importance of the CBS-pair domain in mediating ARL15-dependent suppression of TRPM7 channel activity. To confirm this result, we used the previously identified CNNM2 CBS-pair domain mutants H523K and F524K, which disrupt its binding to ARL15 (61). In wild-type HEK-293 cells, co-expression of ARL15 with WT CNNM2 blocks Mg^2+^-efflux; whereas it is ineffective in interfering with Mg^2+^-efflux mediated by the CNNM2-H523K and CNNM2-F524K mutants (Fig. 7*C*). Loss of CNNM-binding similarly disrupted ARL15-mediated inhibition of TRPM7 channel activity. Co-expression in 293-M7-ΔCNNM3/4 cells of WT ARL15, CNNM2 containing both the S269P and the H523K mutations (CNNM2-H523K/S269P), and TRPM7 failed to reduce TRPM7 channel current amplitudes (Fig. 7, *D* and *E*). Collectively, these findings demonstrate that ARL15 acts through the CNNM2 CBS-pair domain to modulate the TRPM7 channel.

## Discussion

The purpose of this study was to advance our understanding of the molecular basis for CNNMs' regulation of TRPM7. Our biochemical mapping experiments demonstrated that the CNNM2's NH_2_-terminus, which contains the transmembrane domain, is sufficient for complex assembly with TRPM7. Using GST pull-down assays, we identified additional interaction sites between the cytoplasmic domains of TRPM7 and CNNM2. Specifically, the CBS-pair domain of CNNM2 bound to both the NH_2_- and COOH-terminal regions of TRPM7, whereas the CNBH domain selectively interacted with the COOH-terminal region. To further delineate these interactions, we mapped binding sites within TRPM7's COOH-terminal region and found that the CBS-pair domain interacts with the serine/threonine rich region, which undergoes extensive autophosphorylation by TRPM7's kinase (78). In contrast, the CNBH domain interacts with both the ST-region of TRPM7 and its catalytic kinase domain. A prior study reported that co-expression of CNNM3 with TRPM7 suppresses TRPM7's kinase activity, as evidenced by reduced autophosphorylation on Ser1511 (60). To assess whether CNNM2 modulates kinase activity, we conducted *in vitro* kinase assays using purified GST-CBS and GST-CNBH and the SUMO-tagged TRPM7 ST-kinase. While the CBS-pair domain had no measurable effect, the CNBH domain of CNNM2 modestly enhanced TRPM7 kinase activity. Notably, TRPM7's kinase phosphorylates the CNBH domain *in vitro*. These findings suggest that CNNMs may influence TRPM7 autophosphorylation and themselves serve as kinase substrates. Future studies will investigate the biological relevance of these phosphorylation events. Additional work is also required to define the function of the TRPM7 kinase in regulating the CNNM-TRPM7 complex, how kinase activity is controlled by CNNMs, and whether ARL15 and PTP4A proteins modulate this regulation.

Using whole-cell electrophysiology, we observed that expression of CNNM4-S196P and CNNM2-S269P-mutants with disabled Mg^2+^-export activity increased TRPM7 channel activity when co-expressed with TRPM7 in 293-M7-ΔCNNM3/4 cells. Co-expression of these mutants did not affect TRPM7's inward or outward current amplitudes. As both mutants have disabled Mg^2+^-efflux activity, these results suggest that CNNMs can regulate TRPM7 independent of their Mg^2+^-export function. Nonetheless, it remains possible that CNNM2 and CNNM4, when Mg^2+^-export competent, may further potentiate channel activation by lowering local intracellular Mg^2+^ concentrations in the immediate vicinity of TRPM7's inter-subunit Mg^2+^ regulatory site (N1097), which is located at the channel's lower gate (57). Interestingly, co-expression of CNNM2 or CNNM4 lacking the CBS-pair domain with TRPM7 also enhanced channel activity in 293-M7-ΔCNNM3/4 cells. In contrast, the CNNM2-T568I mutant, which stabilizes the CBS-pair domain in a flat, “Mg·ATP”-bound form conformation failed to augment TRPM7 channel activity. Nonetheless, we did not observe differential inhibitory effects of Mg·ATP on CNNM2-S269P *versus* CNNM2-ΔCBS, suggesting that the regulatory effects of the CBS-pair domain occur independently of Mg·ATP binding.

**Figure 8:**
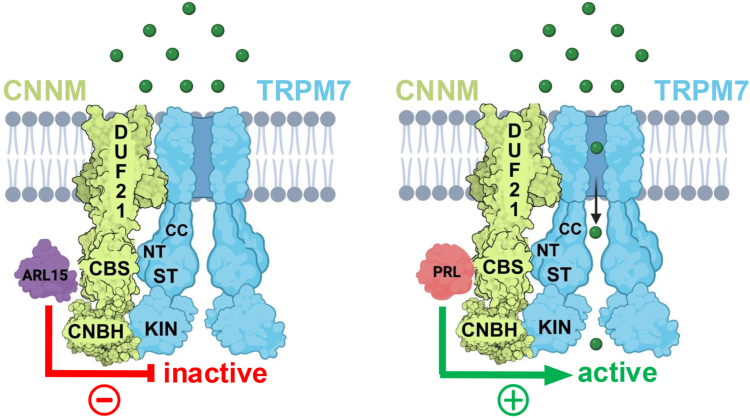
**Model of the CNNM-TRPM7 complex.** CNNMs bind to TRPM7 to modulate its channel-kinase activities. Shown is an artistic rendering of the CNNM-TRPM7 complex in the “inactive” and “active” states; no structural significance is implied. Extracellular domains of CNNMs and TRPM7 have been removed to ease visualizing. Our experiments indicate that CNNMs' NH_2_-terminal region, which contains the DUF21 transmembrane domain, plays a key role in anchoring CNNMs to the TRPM7 channel. Additional weaker interactions occur between CNNM2's CBS-pair domain and TRPM7's NH_2_-terminus (NT) and COOH-terminal region containing the coiled-coil (CC) domain, Serine-threonine rich region (ST), and TRPM7 catalytic kinase (KIN) domain. The CNBH domain of CNNM2 does not interact with the TRPM7 NH_2_-terminal domain but binds to the kinase domain, modestly stimulating kinase activity. The CNBH domain itself is also subject to phosphorylation by the TRPM7 kinase. Our data highlight the essential role for the CNNM CBS-pair domain in mediating CNNM-dependent modulation of TRPM7. ARL15, which binds to the CBS-pair domain of CNNMs, inactivates TRPM7. In contrast, PRLs also bind to CNNMs' CBS-pair domain but enhance TRPM7 channel activity-potentially by displacing ARL15. ARL15 and PRL compete for binding to CNNMs CBS domain, suggesting that functional state of the CNNM-TRPM7 complex may be governed by the relative expression levels of the regulatory proteins. The precise molecular mechanism(s) by which ARL15 and PRLs regulate TRPM7 remain unresolved, but may involve changes in channel gating, altered protein expression or glycosylation, and/or changes in trafficking of TRPM7 to the cell's plasma membrane. The effect of ARL15 and PRLs on TRPM7 kinase activity remains to be determined.

To further explore this hypothesis, we examined whether the CNNM binding protein ARL15 regulates TRPM7 *via* the CBS-pair domain. Previous studies showed that ARL15 suppressed TRPM7 channel function when overexpressed (60), while knockdown of ARL15 enhances TRPM7 function, supporting a role for endogenous ARL15 in channel regulation. Since ARL15 binds directly to the CNNM2 CBS-pair domain (61), we tested whether this domain is required for ARL15-dependent inhibition. Deletion of the CBS-pair domain or the H523K mutation in CNNM2, which abolishes ARL15 binding (61), both disrupted ARL15's ability to inhibit TRPM7 channel activity. This data supports a model in which the CBS-pair domain of CNNMs functions as a signaling hub through which CNNM-binding partners modulate the divalent-influx capacity of the CNNM-TRPM7 complex (Fig. 8). In this model, CNNMs' NH_2_-terminus, containing the transmembrane domain, mediates assembly with TRPM7, and the CBS pair domain regulates channel function. Although ARL15 clearly inhibits TRPM7 channel function, it remains unclear whether PRLs directly stimulate the channel or act by displacing ARL15. Finally, although the CNBH domain of CNNMs enhance TRPM7 kinase activity, the mechanisms by which this occurs remains to be elucidated.

The three-dimensional structure of murine TRPM7, truncated to exclude the COOH-terminal serine-threonine (ST)-rich region and kinase domain (a.a. 1–1280) has been determined using cryogenic electron microscopy (87). In this structure, the tetrameric TRPM7 surrounds a central ion-conducting pore that feeds into a 50 Å × 50 Å cytoplasmic space created by a ∼100-Å-tall, ∼120-Å-wide intracellular skirt. This intracellular skirt is composed of TRPM7's NH_2_- MHD and COOH-terminal coiled-coil domains (87). Structures of TRPM7 in the open and closed states have also been reported (88). Although the positions of the first four spanning transmembrane helices (S1–S4) and coiled-coil domains in TRPM7 remain unchanged between these two states, key differences are observed in the pore domain, particularly in the orientation of the TRP helices. The movement of the TRP helices is further propagated to the N-terminal domains, which move as rigid bodies away from the channel's four-fold rotational axis (88). Notably, the, the ST-region and kinase domain of TRPM7-regions where we identified interactions with the CBS-pair and CNBH domains of CNNM2-were absent from these structural models of TRPM7, which were truncated at amino acid 1230, just beyond the coiled-coil domain. As such, it remains unclear whether binding of ARL15 or PRLs to CNNM2's CBS-pair domain induces structural changes that influence the positioning of the TRP helices or other gating elements of TRPM7. This uncertainty underscores the need for additional structural studies incorporating the full-length TRPM7 protein and its regulatory binding partners. It is also possible that the CBS-pair domain does not directly regulate TRPM7 through conformational changes affecting channel gating. Instead, alternative mechanisms such as altered membrane trafficking or changes in protein stability may contribute to the observed regulation. Nevertheless, a clear picture is beginning to emerge in which ARL15, PRLs and CNNMs function together to modulate TRPM7 complexes of different compositions, thereby fine-tuning control of the channel's ever-expanding biological roles.

## Experimental procedures

### Plasmids

The plasmid for expression of murine HA-tagged TRPM7 (TRPM7-WT) in pcDNA5/FRT-TO vector was previously described (51). The kinase-inactive version of HA-TRPM7 (TRPM7-KI) containing the (K1646R) mutation is also in the pCDNA5/FRT-TO vector and was created and used in earlier studies (75, 81). Plasmids for expression of WT and kinase-inactive (K1646R) COOH-terminal fragments of TRPM7 fused to the mfGFP tag (1120–1862), M7-CTERM and M7-CTERM-KI were created by subcloning these fragments from the pcDNA6-V5-HisB vector (89). First, the mfGFP-M7-CTERM and mfGFP-M7-CTERM-KI expression cassettes (HindIII/BamHI fragments) were excised and then ligated into pcDNA5-FRT-TO. Plasmids for expressions of M7-ST-KIN (1288–1862) and M7-KIN (1510–1862) were similarly made, subcloning HindII/BamHI fragments containing mfGFP-M7-ST-KIN and mfGFP-M7-KIN into the pcDNA5-FRT-TO vector. The plasmid for expression of mfGFP-ST was made using QuikChange Mutagenesis to delete the catalytic kinase domain from pcDNA5-FRT-TO containing mfGFP-M7_ST-KIN (1288–1594; see Table S1 for a description of the primers). Likewise, the plasmid expressing M7-NTERM (1–757) was made using QuikChange Mutagenesis (Agilent) to delete amino acids 758 to 1862 in the pcDNA5-FRT-TO-HA-TRPM7 vector (Table S1). FLAG-tagged mouse CNNM2, human CNNM3, and human CNNM4 expression plasmids in pCMV-4A were previously described (72) and received as a gift from Dr Hiroaki Miki (Osaka University). The following CNNM2 fragments were subcloned by PCR (see Table S1 for primer information) into the EcoRI and HindIII sites of pCMV-4A in frame with the FLAG tag: CNNM2 NH_2_-terminal domain (CNNM2-NTERM (CNNM2-NT) (1–402), CNNM2 lacking the CNBH domain (CNNM2-ΔCNBH(1–582), CNNM2 CBS domain (CBS; 402–582), and CNNM2 CNBH domain (CNBH; 582–875). CNNM2 and CNNM3 lacking the tandem-CBS domains (CNNM2-ΔCBS(469–578) and CNNM3-ΔCBS(367–451)) as well as CNNM2-T568I were made using QuikChange Mutagenesis (Agilent, Santa Clara, CA; See Table S1 for primer information). FLAG-, His-, and SUMOSTAR-tagged CNBH and CBS were synthesized by Genescript and subcloned into the NheI/EcorI sites of the pcDNA3.1(+) vector. Origene provided expression plasmids for Myc-DDK tagged ARL15 in the pCMV6-Entry vector. Plasmids for expressing GST-fusion proteins containing the CNBH domain (582–875) and CBS domain (495–582) of CNNM2 were codon optimized for bacterial expression and synthesized by GenScript, and subcloned into the EcoRI/XhoI sites of the pGEX-6P3 vector. CNNM4-ΔCBS(396–505), and CNNM4-S196P in the pCMV-4A vector were previously described (59), as were also the plasmid expressing mouse CNNM2 (residues 1–875)-mCherry-HA and CBS domain mutants (Mahbub *et al.*, 2023). HA-tagged CNNM2-WT and HA-tagged CNNM2-NT were made by PCR-directed cloning (see Table S1 for primer information) into the pcDNA3-neo-cterminal-3HA vector. pcDNA3-neo-cterminal-3HA was a gift from Michael E. Wright (Addgene plasmid # 102643; http://n2t.net/addgene:102643; RRID:Addgene_102643). HA-tagged SLC41A1 and FLAG-tagged TRPM2 were a gift from Andrew Scharenberg.

### Cell lines

The HEK-293T cell line (CRL-3216) was purchased from The American Type Culture Collection (ATCC). The LTRPC7 cell line has been previously described (1) and in this paper is referred to as 293-TRPM7 cells. 293-TRPM7 cells express FLAG-tagged mouse TRPM7 under tetracycline control was generously provided by Dr Andrew Scharenberg (University of Washington). 293-TRPM7 cells were previously validated by Western blotting for tetracycline-induced expression of TRPM7 (data not shown). All cells were maintained in a humidified 37 °C, 5% CO_2_ incubator using Dulbecco's Modified Eagle Medium, a high-glucose media with 10% fetal bovine serum. 293-TRPM7 cells were plated on poly-L-lysine-coated plates to prevent cell detachment brought on by TRPM7 overexpression. The Zn^2+^ imaging experiments were carried out using the HEK-293T and the LTRPC7 cell lines. Transient transfections were carried out for biochemical assays using the Turbofect transfection reagent (ThermoFisher) following the manufacturer's instructions. The LTRPC7 cells lines expressing TRPM7 and deficient in CNNM3 and CNNM4 (293-M7-ΔCNNM3/4 cells) were previously described (59). 293-M7-ΔCNNM3/4 cells were validated by Western blotting for tetracycline-induced expression of TRPM7 and loss of protein expression of CNNM3 and CNNM4 (59). All cell lines have been tested for *mycoplasma* and were free of mycoplasm for all experiments.

### Purification of SUMO-tagged-ST-KINASE (SUMO-ST-KIN), GST, GST-CBS, and GST-CNBH

SUMO-ST-KIN was expressed in transformed *E. Coli* BL21-DE3 cells (Stratagene, CA) as previously described (81). Bacteria were grown at 37 °C to OD600 of 0.6 to 0.8, cooled to 16 °C, and induced at 16 °C for 16 to 18 h with 1 mM isopropyl-beta-D-thiogalactopyranoside (Gold Biotechnology). Cells were lysed by sonication in ice-cold phosphate-buffered saline (PBS) containing a final concentration of 1 mM protease inhibitor PMSF and 1% Triton-X10. SUMO-TRPM7-Kinase was pulled down from the cell lysate supernatant by streptavidin agarose (Agilent) and eluted with 10 mM Biotin (Millipore Sigma). GST, GST-CBS, and GST-CNBH were expressed in transformed *E. Coli* BL21-DE3 cells. Bacteria were grown at 37 °C to OD600 of 0.6 to 0.8 and induced at 37 °C for 4 to 6 h with 1 mM isopropyl-beta-D-thiogalactopyranoside (Gold Biotechnology). Cells were lysed by sonication in ice-cold phosphate-buffered saline (PBS) containing a final concentration of 1 mM protease inhibitor PMSF. GST, GST-CBS, and GST-CNBH were purified from bacterial cell lysates by incubation with glutathione agarose (Sigma–Aldrich) overnight at 4 °C with rotation, followed by column purification and elution using buffer containing 10 mM Tris, pH 8.0, and 10 mM glutathione. For GST pull-down assays, bound GST proteins on glutathione agarose were washed three times with PBS and then resuspended as a 50% slurry with PBS.

### TRPM7 kinase assay

TRPM7 kinase assay was performed as described (81). Briefly, purified SUMO-ST-M7, GST, GST-CBS, and GST-CNBH proteins were dialyzed into the following buffer (50 mM MOPS (pH 7.2), 100 mM NaCl). The kinase reactions were performed at 30 °C for various times in the presence of 4 μCi of [γ-^32^P]ATP with 5 μg of myelin basic protein (MBP) as a generic substrate in kinase buffer (50 mM MOPS (pH 7.2), 100 mM NaCl, 2.5 mM MnCl_2_, 0.5 mM ATP). Reactions were stopped by the addition of SDS sample buffer, and the reaction mix was resolved by SDS-PAGE. Proteins were detected by Coomassie blue staining and gels were dried completely using a gel dryer (BioRad Laboratories). The incorporation of ^32^P into substrates was analyzed by autoradiography using Cyclone Plus Phosphor Imager (PerkinElmer).

### Identification of TRPM7 kinase phosphorylation sites on the GST-CNBH domain

100 nmol protein were denatured with 2M urea, 0.5% formic acid and 10 mM TCEP at room temperature for 30 min. On-line digestion and subsequence LC-MSMS were conducted using vanquish UPLC coupled to Q-Exactive HF (ThermoFisher). The sample was first online digested with a protease type XVIII/pepsin column (NovaBioassays). The digested peptides were trapped and washed on a self-packed Poros R10 (ABSciex), 2.1 × 4cm trap column with 0.2% formic acid at flowrate of 100 ul/min. Peptides were separated on a 2.1 mm × 5 cm C18 column (1.9 μ, Hypersil Gold, Thermofisher) with a multistep gradient (0–4 min: 2%B, from 2% B to 10% B in 0.3 min,: 10–40%B in 7.6 min (A: 0.2% formic acid, B: 0.1% formic acid in acetonitrile)) at flowrate of 150 ul/min. The mass was measured with resolution of 120,000 and mass range from 300 to 2000 with top 20 peaks fragmented by HCD (relative collision energy 27) and scanned with resolution 15,000 with dynamic exclusion for 20 s.

The LC-MSMS data were analyzed using Proteome Discoverer 3.1 with Sequest HT against custom database composed of target proteins and kinases. MS window was set at ± 10 ppm, MS/MS window set at ± 0.02Da. N-terminal acetylation was added as dynamic protein terminus modification, oxidation at methionine, phosphorylation at serine/threonine/tyrosine were set as dynamic modifications. Protease was set as non-specific. The results were validated by Fix Value PSM Validator with max Delta CN < 0.05. PhosphoRS was used to calculate phosphorylation site confidence. The phosphorylation sites were further manually validated. To quantify the extent of phosphorylation on a particular site, peptide abundance (peak area) of all peptides with the phosphorylated site were tallied against the summed abundance of all peptides containing the site regardless of modifications.

### Immunocytochemistry

293-TRPM7 cells stably expressing tetracycline-inducible HA-tagged TRPM7 were plated on glass coverslips and transfected with FLAG-CNNM2-WT, FLAG-CNNM2-ΔCBS or FLAG-CNNM2-ΔCNBH, or FLAG- CNNM2-NT. Cells were fixed with 4% formaldehyde at room temperature for 30 min for 24 h after transfection. The cells were then permeabilized with phosphate-buffered saline (PBS) containing 0.1% Triton at 30°C for 20 min. The cells were then blocked with 5% FBS/PBS at 30°C for an hour. HA-tagged TRPM7 was detected using a rat monoclonal anti-HA antibody (3F10; Roche Life Sciences), and the FLAG-tagged CNNMs were detected using a rabbit anti-FLAG M2 antibody (Cell Signaling). The secondary antibodies used were goat antibodies produced against rat and rabbit, labeled with Alexa FluorTM 488 and Alexa FluorTM 568 (ThermoFisher). Images were captured using a Yokogawa CSUX1-5000 microscope at the Rutgers RWJMS CORE Confocal facility, utilizing 488 nm and 561 nm excitation wavelengths.

### GST pull-down assay

GST, GST-CBS, and GST-CNBH were expressed in *E. Coli* BL21-DE3 cells (Agilent). Sonication was used to lyse bacterial cells in ice-cold PBS containing 1% Triton X-100 and the protease inhibitor phenylmethylsulfonyl fluoride (Millipore Sigma). The bacterial cell lysates were then incubated/rotated overnight at 4 °C with Glutathione agarose (Millipore Sigma). PBS containing 1% Triton X-100 was used to wash the Glutathione agarose beads. For the GST pull-down assay, a 10 cm dish containing HEK-293T cells at 80% confluence was transiently transfected with 10 μg of the plasmids for expression of M7-CTERM, M7-CTERM-KI, M7-ST-KIN, M7-KIN, and M7-ST plasmids. The cells were lysed with 1 ml of mild lysis buffer containing a protease inhibitor mixture (ThermoFisher), and spun down at 14,000*g* for 10 min at 4 °C. Supernatants from the cell lysates were incubated with Glutathione agarose beads bound to either GST, GST-CBS, or GST-CNBH proteins overnight at 4 °C. The bound proteins were washed three times with 1 ml of PBS containing 0.1% Triton X-100 and eluted into 50 μl of SDS sample buffer. Lysates from input (10–20 μl) and pull-down samples (15–20 μl) were resolved by SDS-PAGE gels and Western blotting. SDS-PAGE and Coomassie staining were used to visualize the GST-fusion proteins used in the assay.

### Western blotting

Standard methods for SDS-PAGE and Western blotting were used to resolve the proteins for protein detection. The rat monoclonal anti-HA antibody (3F10; Roche Life Sciences) was utilized to identify HA-tagged TRPM7. The FLAG-tagged CNNMs and ARL15 were identified using an anti-FLAG antibody (MilliporeSigma or Cell Signaling Technology). M7-CTERM-KI, M7-ST-KIN, M7-KIN, and M7-ST were identified using the rabbit polyclonal anti-TRPM7-C47, which was previously described (56). PRL1 and PRL2 were detected using a mouse monoclonal anti-PTP4A (PRL2 clone 42) antibody (Millipore Sigma). Vinculin was detected using a mouse anti-Vinculin antibody (Millipore Sigma). β-actin was detected using the mouse monoclonal anti-β-actin antibody sc-47778 (Santa Cruz Biotechnology). Protein quantification utilizing the Pierce BCA Protein Assay Kit (ThermoFisher) was used to measure protein concentrations according to the manufacturer's protocol. An Amersham Imager 680 was used to capture blot images developed with horseradish peroxidase-conjugated secondary antibodies using Thermo Scientific SuperSignal West Pico PLUS Chemiluminescent Substrate.

### Immunoprecipitation

For immunoprecipitation, a 10 cm dish containing HEK-293T cells at 80% confluence was transiently transfected with the following plasmids and the proteins allowed to express for 48 h: FLAG-tagged WT CNNM2, CNNM2-NTERM, CNNM2-CTERM, CNNM2-ΔCBS, CNNM2-ΔCNBH, SUMO-CBS, SUMO-CNBH (3–3.5 μg), and HA-tagged TRPM7 or an empty vector control pcDNA5-FRT-TO (7.0 μg). The cells were lysed with 1 ml of mild lysis buffer containing a protease inhibitor mixture (ThermoFisher), and spun down at 14,000*g* for 10 min at 4 °C. Supernatants from the cell lysates were incubated with anti-HA magnetic beads (ThermoFisher) to immunoprecipitate HA-TRPM7 from cell lysates overnight at 4 °C. The bound proteins were washed three times with 1 ml of PBS containing 0.1% Triton X-100 and eluted into 50 μl of SDS sample buffer. Lysates from input (20 μl) and pull-down samples (20 μl) were resolved by SDS-PAGE gels and Western blotting. SDS-PAGE and Coomassie staining were used to visualize the GST-fusion proteins used in the assay. A rabbit anti-FLAG M2 antibody was utilized to detect FLAG-CNNMs (Cell Signaling Technology), whereas a rat monoclonal anti-HA-antibody was used to detect TRPM7 (3F10; Roche Life Sciences).

### Magnesium Green Mg^2+^ efflux assay

HEK-293T cells were grown in a 3.5 cm glass bottom dish with a poly-lysine coating to ensure adherence. Cells were transfected with mCNNM2-mCherry fusion proteins (WT and mutants) and FLAG-tagged ARL15 at a ratio of 1:3, mCNNM2-mCherry:ARL15. To ensure good cell separation, transfection was performed at densities between 20% and 30% using Lipofectamine 3000. Transfected cells were grown for 24 h in DMEM media supplemented with 40 mM MgCl_2_. Cells were then incubated in Mg^2+^ loading buffer (78.1 mM NaCl, 5.4 mM KCl, 1.8 mM CaCl_2_, 40 mM MgCl_2_, 5.5 mM glucose, 5.5 mM HEPES, pH to 7.4 with KOH) supplemented with 2 mM Magnesium Green dye (Fisher Scientific) and 0.025% Pluronic f-127 (Fisher Scientific) in DMSO for 30 min at 37 °C. Following incubation, the cells were rinsed once with the Mg^2+^ loading buffer at room temperature and viewed *via* microscopy (AxioObserver.Z1 with X-cite series 120Q Illuminator, AxioCamMR3 camera, under the control of Axiovision software). Magnesium Green fluorescence (excitation 450–488 nm and emission 500–548 nm) was measured for 5 min (Filter set ET-GFP 49002, objective ET Plan-Neofluor 10 × /0.20 Ph 1) with 20 s between frames. At 1 min, the buffer was changed to Mg^2+^-free buffer (138.1 mM NaCl, 5.4 mM KCl, 1.8 mM CaCl_2_, 5.5 mM glucose, 5.5 mM HEPES, pH to 7.4 with KOH). Following the efflux assay, CNNM2-mCherry expression was detected by measuring fluorescence from the ET-mCherry Texas Red 49,008 filter set (excitation 540–580 nm and emission 590–670 nm). Cells were then fixed by 3.7% formaldehyde and FLAG-tagged ARL15 proteins detected with anti-FLAG antibody (1:2000, F7425, Sigma-Aldrich) and Alexa 488-conjugated goat anti-rabbit secondary antibodies (A-11034, ThermoFisher). Fluorescence in 10 cells expressing the proteins of interest was quantified using Fiji analysis software and the average plotted as function of time.

### Electrophysiological recordings

The voltage-clamp technique was used to evaluate the whole-cell currents of TRPM7 expressed in 293-M7-ΔCNNM3/4 cells as previously described (Li *et al.*, 2007). Briefly, whole-cell current recordings of TRPM7-expressing cells were elicited by voltage stimuli lasting 250 ms delivered every 1 s using voltage ramps from −100 to +100 mV. Data were digitized at two or 5 kHz and digitally filtered offline at 1 kHz. The internal pipette solution for macroscopic current recordings contained 145 mM Cs methanesulfonate, 8 mM NaCl, 10 mM EGTA, and 10 mM HEPES, pH adjusted to 7.2 with CsOH. The extracellular solution for whole-cell recordings contained 140 mM NaCl, 5 mM KCl, 2 mM CaCl_2_, 10 mM HEPES, and 10 mM glucose, pH adjusted to 7.4 with NaOH. To assay the impact of the CNNM variants on TRPM7 channel activity, CNNMs were transiently transfected into 293-M7-ΔCNNM3/4 cells using Lipofectamine 3000 reagent. Tetracycline was added to the growth media, and whole-cell electrophysiological recordings were performed between 12 and 30 h after tetracycline induction for protein expression.

### Statistical analysis

Statistical analysis was performed using GraphPad Prism version 10.0.1 (218), as described in the figure legends. A *p*-value of <0.05 was considered significant. Error bars indicate standard error. Individual biological replicates are shown in electrophysiology and kinase assay experiments.

## Data availability

All data is contained within the manuscript.

## Supporting information

This article contains supporting information.

## Conflict of interest

The authors declare that they have no conflicts of interest with the contents of this article.

## References

[1] Nadler M.J., Hermosura M.C., Inabe K., Perraud A.L., Zhu Q., Stokes A.J. (2001). LTRPC7 is a Mg.ATP-regulated divalent cation channel required for cell viability. Nature.

[2] Runnels L.W., Yue L., Clapham D.E. (2001). TRP-PLIK, a bifunctional protein with kinase and ion channel activities. Science.

[3] Monteilh-Zoller M.K., Hermosura M.C., Nadler M.J., Scharenberg A.M., Penner R., Fleig A. (2003). TRPM7 provides an ion channel mechanism for cellular entry of trace metal ions. J. Gen. Physiol..

[4] Bernhardt M.L., Stein P., Carvacho I., Krapp C., Ardestani G., Mehregan A. (2018). TRPM7 and Ca(V)3.2 channels mediate Ca(2+) influx required for egg activation at fertilization. Proc. Natl. Acad. Sci. U. S. A..

[5] Jin J., Desai B.N., Navarro B., Donovan A., Andrews N.C., Clapham D.E. (2008). Deletion of Trpm7 disrupts embryonic development and thymopoiesis without altering Mg2+ homeostasis. Science.

[6] Jin J., Wu L.J., Jun J., Cheng X., Xu H., Andrews N.C. (2012). The channel kinase, TRPM7, is required for early embryonic development. Proc. Natl. Acad. Sci. U. S. A..

[7] Komiya Y., Bai Z., Cai N., Lou L., Al-Saadi N., Mezzacappa C. (2017). A nonredundant role for the TRPM6 channel in neural tube closure. Sci. Rep..

[8] Liu W., Su L.T., Khadka D.K., Mezzacappa C., Komiya Y., Sato A. (2011). TRPM7 regulates gastrulation during vertebrate embryogenesis. Dev. Biol..

[9] Ryazanova L.V., Rondon L.J., Zierler S., Hu Z., Galli J., Yamaguchi T.P. (2010). TRPM7 is essential for Mg(2+) homeostasis in mammals. Nat. Commun..

[10] Schutz A., Richter C., Weissgerber P., Tsvilovskyy V., Hesse M., Ottenheijm R. (2021). Trophectoderm cell failure leads to peri-implantation lethality in Trpm7-deficient mouse embryos. Cell Rep..

[11] Mittermeier L., Demirkhanyan L., Stadlbauer B., Breit A., Recordati C., Hilgendorff A. (2019). TRPM7 is the central gatekeeper of intestinal mineral absorption essential for postnatal survival. Proc. Natl. Acad. Sci. U. S. A..

[12] Elizondo M.R., Arduini B.L., Paulsen J., MacDonald E.L., Sabel J.L., Henion P.D. (2005). Defective skeletogenesis with kidney stone formation in dwarf zebrafish mutant for trpm7. Curr. Biol..

[13] Elizondo M.R., Budi E.H., Parichy D.M. (2010). trpm7 regulation of in vivo cation homeostasis and kidney function involves stanniocalcin 1 and fgf23. Endocrinology.

[14] Low S.E., Amburgey K., Horstick E., Linsley J., Sprague S.M., Cui W.W. (2011). TRPM7 is required within zebrafish sensory neurons for the activation of touch-evoked escape behaviors. J. Neurosci..

[15] McNeill M.S., Paulsen J., Bonde G., Burnight E., Hsu M.Y., Cornell R.A. (2007). Cell death of melanophores in zebrafish trpm7 mutant embryos depends on melanin synthesis. J. Invest. Dermatol..

[16] Mudassir B.U., Agha Z. (2023). Novel biallelic frameshift in TRPM7 gene causes Hallermann-Streiff syndrome in a consanguineous family: a case report. Acta Neurol. Belg..

[17] Stritt S., Nurden P., Favier R., Favier M., Ferioli S., Gotru S.K. (2016). Defects in TRPM7 channel function deregulate thrombopoiesis through altered cellular Mg(2+) homeostasis and cytoskeletal architecture. Nat. Commun..

[18] Bosman W., Butler K.M., Chang C.A., Ganapathi M., Guzman E., Latta F. (2024). Pathogenic heterozygous TRPM7 variants and hypomagnesemia with developmental delay. Clin. Kidney J..

[19] Middelbeek J., Kuipers A.J., Henneman L., Visser D., Eidhof I., van Horssen R. (2012). TRPM7 is required for breast tumor cell metastasis. Cancer Res..

[20] Visser D., Langeslag M., Kedziora K.M., Klarenbeek J., Kamermans A., Horgen F.D. (2013). TRPM7 triggers Ca2+ sparks and invadosome formation in neuroblastoma cells. Cell Calcium.

[21] Yankaskas C.L., Bera K., Stoletov K., Serra S.A., Carrillo-Garcia J., Tuntithavornwat S. (2021). The fluid shear stress sensor TRPM7 regulates tumor cell intravasation. Sci. Adv..

[22] Du J., Xie J., Zhang Z., Tsujikawa H., Fusco D., Silverman D. (2010). TRPM7-mediated Ca2+ signals confer fibrogenesis in human atrial fibrillation. Circ. Res..

[23] Rios F.J., Zou Z.G., Harvey A.P., Harvey K.Y., Camargo L.L., Neves K.B. (2022). TRPM7 deficiency exacerbates cardiovascular and renal damage induced by aldosterone-salt. Commun. Biol..

[24] Suzuki S., Penner R., Fleig A. (2020). TRPM7 contributes to progressive nephropathy. Sci. Rep..

[25] Busey G.W., Manjegowda M.C., Huang T., Iobst W.H., Naphade S.S., Kennedy J.A. (2023). Novel TRPM7 inhibitors with potent anti-inflammatory effects in vivo. bioRxiv.

[26] Hoeger B., Nadolni W., Hampe S., Hoelting K., Fraticelli M., Zaborsky N. (2023). Inactivation of TRPM7 kinase targets AKT signaling and Cyclooxygenase-2 expression in human CML cells. Function (Oxf).

[27] Zhong W., Ma M., Xie J., Huang C., Li X., Gao M. (2023). Adipose-specific deletion of the cation channel TRPM7 inhibits TAK1 kinase-dependent inflammation and obesity in male mice. Nat. Commun..

[28] Aarts M., Iihara K., Wei W.L., Xiong Z.G., Arundine M., Cerwinski W. (2003). A key role for TRPM7 channels in anoxic neuronal death. Cell.

[29] Sun H.S., Jackson M.F., Martin L.J., Jansen K., Teves L., Cui H. (2009). Suppression of hippocampal TRPM7 protein prevents delayed neuronal death in brain ischemia. Nat. Neurosci..

[30] Zhang P., Li W., Liu Y., Gao Y., Abumaria N. (2022). Neuroprotective effects of TRPM7 deletion in parvalbumin GABAergic vs. glutamatergic neurons following ischemia. Cells.

[31] Wang X., Wang M., Zhu T.T., Zheng Z.J., Li S., Sui Z.Y. (2025). The TRPM7 chanzyme in smooth muscle cells drives abdominal aortic aneurysm in mice. Nat. Cardiovasc. Res..

[32] Zong P., Li C.X., Feng J., Yue Z., Nethramangalath T., Xie Y. (2025). TRPM7 channel activity promotes the pathogenesis of abdominal aortic aneurysms. Nat. Cardiovasc. Res..

[33] Desai B.N., Krapivinsky G., Navarro B., Krapivinsky L., Carter B.C., Febvay S. (2012). Cleavage of TRPM7 releases the kinase domain from the ion channel and regulates its participation in Fas-induced apoptosis. Dev. Cell.

[34] Schappe M.S., Szteyn K., Stremska M.E., Mendu S.K., Downs T.K., Seegren P.V. (2018). Chanzyme TRPM7 mediates the Ca(2+) influx essential for lipopolysaccharide-induced toll-like receptor 4 endocytosis and macrophage activation. Immunity.

[35] Jiang Z.J., Li W., Yao L.H., Saed B., Rao Y., Grewe B.S. (2021). TRPM7 is critical for short-term synaptic depression by regulating synaptic vesicle endocytosis. Elife.

[36] Schappe M.S., Stremska M.E., Busey G.W., Downs T.K., Seegren P.V., Mendu S.K. (2022). Efferocytosis requires periphagosomal Ca(2+)-signaling and TRPM7-mediated electrical activity. Nat. Commun..

[37] Khajavi N., Beck A., Ricku K., Beyerle P., Jacob K., Syamsul S.F. (2023). TRPM7 kinase is required for insulin production and compensatory islet responses during obesity. JCI Insight.

[38] Sahni J., Scharenberg A.M. (2008). TRPM7 ion channels are required for sustained phosphoinositide 3-kinase signaling in lymphocytes. Cell Metab..

[39] Sahni J., Tamura R., Sweet I.R., Scharenberg A.M. (2010). TRPM7 regulates quiescent/proliferative metabolic transitions in lymphocytes. Cell Cycle.

[40] Wu W., Wang X., Liao L., Chen J., Wang Y., Yao M. (2023). The TRPM7 channel reprograms cellular glycolysis to drive tumorigenesis and angiogenesis. Cell Death Dis..

[41] Schmitz C., Perraud A.L., Johnson C.O., Inabe K., Smith M.K., Penner R. (2003). Regulation of vertebrate cellular Mg2+ homeostasis by TRPM7. Cell.

[42] Clark K., Langeslag M., van Leeuwen B., Ran L., Ryazanov A.G., Figdor C.G. (2006). TRPM7, a novel regulator of actomyosin contractility and cell adhesion. EMBO J..

[43] Faouzi M., Kilch T., Horgen F.D., Fleig A., Penner R. (2017). The TRPM7 channel kinase regulates store-operated calcium entry. J. Physiol..

[44] Gotru S.K., Chen W., Kraft P., Becker I.C., Wolf K., Stritt S. (2018). TRPM7 Kinase controls calcium responses in arterial thrombosis and stroke in mice. Arterioscler Thromb. Vasc. Biol..

[45] Langeslag M., Clark K., Moolenaar W.H., van Leeuwen F.N., Jalink K. (2007). Activation of TRPM7 channels by phospholipase C-coupled receptor agonists. J. Biol. Chem..

[46] Starostina I., Jang Y.K., Kim H.S., Suh J.S., Ahn S.H., Choi G.H. (2021). Distinct calcium regulation of TRPM7 mechanosensitive channels at plasma membrane microdomains visualized by FRET-based single cell imaging. Sci. Rep..

[47] Wei C., Wang X., Chen M., Ouyang K., Song L.S., Cheng H. (2009). Calcium flickers steer cell migration. Nature.

[48] Abiria S.A., Krapivinsky G., Sah R., Santa-Cruz A.G., Chaudhuri D., Zhang J. (2017). TRPM7 senses oxidative stress to release Zn(2+) from unique intracellular vesicles. Proc. Natl. Acad. Sci. U. S. A..

[49] Krapivinsky G., Krapivinsky L., Manasian Y., Clapham D.E. (2014). The TRPM7 chanzyme is cleaved to release a chromatin-modifying kinase. Cell.

[50] Clark K., Middelbeek J., Lasonder E., Dulyaninova N.G., Morrice N.A., Ryazanov A.G. (2008). TRPM7 regulates myosin IIA filament stability and protein localization by heavy chain phosphorylation. J. Mol. Biol..

[51] Su L.T., Agapito M.A., Li M., Simonson W.T., Huttenlocher A., Habas R. (2006). TRPM7 regulates cell adhesion by controlling the calcium-dependent protease calpain. J. Biol. Chem..

[52] Su L.T., Chen H.C., Gonzalez-Pagan O., Overton J.D., Xie J., Yue L. (2010). TRPM7 activates m-calpain by stress-dependent stimulation of p38 MAPK and c-Jun N-terminal kinase. J. Mol. Biol..

[53] Voringer S., Schreyer L., Nadolni W., Meier M.A., Woerther K., Mittermeier C. (2020). Inhibition of TRPM7 blocks MRTF/SRF-dependent transcriptional and tumorigenic activity. Oncogene.

[54] Demeuse P., Penner R., Fleig A. (2006). TRPM7 channel is regulated by magnesium nucleotides via its kinase domain. J. Gen. Physiol..

[55] Jiang J., Li M., Yue L. (2005). Potentiation of TRPM7 inward currents by protons. J. Gen. Physiol..

[56] Runnels L.W., Yue L., Clapham D.E. (2002). The TRPM7 channel is inactivated by PIP(2) hydrolysis. Nat. Cell Biol..

[57] Schmidt E., Narangoda C., Norenberg W., Egawa M., Rossig A., Leonhardt M. (2022). Structural mechanism of TRPM7 channel regulation by intracellular magnesium. Cell Mol. Life Sci..

[58] Yu H., Zhang Z., Lis A., Penner R., Fleig A. (2013). TRPM7 is regulated by halides through its kinase domain. Cell Mol. Life Sci..

[59] Bai Z., Feng J., Franken G.A.C., Al'Saadi N., Cai N., Yu A.S. (2021). CNNM proteins selectively bind to the TRPM7 channel to stimulate divalent cation entry into cells. PLoS Biol..

[60] Kollewe A., Chubanov V., Tseung F.T., Correia L., Schmidt E., Rossig A. (2021). The molecular appearance of native TRPM7 channel complexes identified by high-resolution proteomics. Elife.

[61] Mahbub L., Kozlov G., Zong P., Lee E.L., Tetteh S., Nethramangalath T. (2023). Structural insights into regulation of CNNM-TRPM7 divalent cation uptake by the small GTPase ARL15. Elife.

[62] Funato Y., Yamazaki D., Mizukami S., Du L., Kikuchi K., Miki H. (2014). Membrane protein CNNM4-dependent Mg2+ efflux suppresses tumor progression. J. Clin. Invest..

[63] Yamazaki D., Funato Y., Miura J., Sato S., Toyosawa S., Furutani K. (2013). Basolateral Mg2+ extrusion via CNNM4 mediates transcellular Mg2+ transport across epithelia: a mouse model PLoS. Genet.

[64] Hardy S., Zolotarov Y., Coleman J., Roitman S., Khursheed H., Aubry I. (2023). PRL-1/2 phosphatases control TRPM7 magnesium-dependent function to regulate cellular bioenergetics. Proc. Natl. Acad. Sci. U. S. A..

[65] de Baaij J.H., Stuiver M., Meij I.C., Lainez S., Kopplin K., Venselaar H. (2012). Membrane topology and intracellular processing of cyclin M2 (CNNM2). J. Biol. Chem..

[66] Bateman A. (1997). The structure of a domain common to archaebacteria and the homocystinuria disease protein. Trends Biochem. Sci..

[67] Baykov A.A., Tuominen H.K., Lahti R. (2011). The CBS domain: a protein module with an emerging prominent role in regulation. ACS Chem. Biol..

[68] Corral-Rodriguez M.A., Stuiver M., Abascal-Palacios G., Diercks T., Oyenarte I., Ereno-Orbea J. (2014). Nucleotide binding triggers a conformational change of the CBS module of the magnesium transporter CNNM2 from a twisted towards a flat structure. Biochem. J..

[69] Hardy S., Uetani N., Wong N., Kostantin E., Labbe D.P., Begin L.R. (2015). The protein tyrosine phosphatase PRL-2 interacts with the magnesium transporter CNNM3 to promote oncogenesis. Oncogene.

[70] Zolotarov Y., Ma C., Gonzalez-Recio I., Hardy S., Franken G.A.C., Uetani N. (2021). ARL15 modulates magnesium homeostasis through N-glycosylation of CNNMs Cell. Mol. Life Sci..

[71] Chen Y.S., Kozlov G., Fakih R., Funato Y., Miki H., Gehring K. (2018). The cyclic nucleotide-binding homology domain of the integral membrane protein CNNM mediates dimerization and is required for Mg(2+) efflux activity. J. Biol. Chem..

[72] Hirata Y., Funato Y., Takano Y., Miki H. (2014). Mg2+-dependent interactions of ATP with the cystathionine-beta-synthase (CBS) domains of a magnesium transporter. J. Biol. Chem..

[73] Kaitsuka T., Katagiri C., Beesetty P., Nakamura K., Hourani S., Tomizawa K. (2014). Inactivation of TRPM7 kinase activity does not impair its channel function in mice. Sci. Rep..

[74] Matsushita M., Kozak J.A., Shimizu Y., McLachlin D.T., Yamaguchi H., Wei F.Y. (2005). Channel function is dissociated from the intrinsic kinase activity and autophosphorylation of TRPM7/ChaK1. J. Biol. Chem..

[75] Cai N., Lou L., Al-Saadi N., Tetteh S., Runnels L.W. (2018). The kinase activity of the channel-kinase protein TRPM7 regulates stability and localization of the TRPM7 channel in polarized epithelial cells. J. Biol. Chem..

[76] Hofmann T., Schafer S., Linseisen M., Sytik L., Gudermann T., Chubanov V. (2014). Activation of TRPM7 channels by small molecules under physiological conditions. Pflugers Arch..

[77] Jansen C., Sahni J., Suzuki S., Horgen F.D., Penner R., Fleig A. (2016). The coiled-coil domain of zebrafish TRPM7 regulates Mg.nucleotide sensitivity. Sci. Rep..

[78] Clark K., Middelbeek J., Morrice N.A., Figdor C.G., Lasonder E., van Leeuwen F.N. (2008). Massive autophosphorylation of the Ser/Thr-rich domain controls protein kinase activity of TRPM6 and TRPM7. PLoS One.

[79] Gimenez-Mascarell P., Oyenarte I., Hardy S., Breiderhoff T., Stuiver M., Kostantin E. (2017). Structural basis of the oncogenic interaction of phosphatase PRL-1 with the magnesium transporter CNNM2. J. Biol. Chem..

[80] Gulerez I., Funato Y., Wu H., Yang M., Kozlov G., Miki H. (2016). Phosphocysteine in the PRL-CNNM pathway mediates magnesium homeostasis. EMBO Rep..

[81] Cai N., Bai Z., Nanda V., Runnels L.W. (2017). Mass spectrometric analysis of TRPM6 and TRPM7 phosphorylation reveals regulatory mechanisms of the channel-kinases. Sci. Rep..

[82] Parry D.A., Mighell A.J., El-Sayed W., Shore R.C., Jalili I.K., Dollfus H. (2009). Mutations in CNNM4 cause Jalili syndrome, consisting of autosomal-recessive cone-rod dystrophy and amelogenesis imperfecta. Am. J. Hum. Genet..

[83] Huang Y., Jin F., Funato Y., Xu Z., Zhu W., Wang J. (2021). Structural basis for the Mg(2+) recognition and regulation of the CorC Mg(2+) transporter. Sci. Adv..

[84] Chen Y.S., Kozlov G., Fakih R., Yang M., Zhang Z., Kovrigin E.L. (2020). Mg(2+)-ATP sensing in CNNM, a putative magnesium transporter. Structure.

[85] Stuiver M., Lainez S., Will C., Terryn S., Gunzel D., Debaix H. (2011). CNNM2, encoding a basolateral protein required for renal Mg2+ handling, is mutated in dominant hypomagnesemia. Am. J. Hum. Genet..

[86] Zhang H., Kozlov G., Li X., Wu H., Gulerez I., Gehring K. (2017). PRL3 phosphatase active site is required for binding the putative magnesium transporter CNNM3. Sci. Rep..

[87] Duan J., Li Z., Li J., Hulse R.E., Santa-Cruz A., Valinsky W.C. (2018). Structure of the mammalian TRPM7, a magnesium channel required during embryonic development. Proc. Natl. Acad. Sci. U. S. A..

[88] Nadezhdin K.D., Correia L., Narangoda C., Patel D.S., Neuberger A., Gudermann T. (2023). Structural mechanisms of TRPM7 activation and inhibition. Nat. Commun..

[89] Overton J.D., Komiya Y., Mezzacappa C., Nama K., Cai N., Lou L. (2015). Hepatocystin is essential for TRPM7 function during early embryogenesis. Sci. Rep..

