# Supplementary material for: The cytoplasmic domains of the CNNM family of transmembrane proteins modulate the ion channel-kinase TRPM7
Source: J Biol Chem. 2025 Sep 15;301(11):110720. doi: 10.1016/j.jbc.2025.110720 (PMC12547303; doi:10.1016/j.jbc.2025.110720)
Supplement: Supporting information [file mmc1.pdf]

Supporting Information for

The cytoplasmic domains of the CNNM family of transmembrane proteins modulate the ion channel-kinase TRPM7

Sandra Tetteh[1], Pengyu Zong[2], Jianlin Feng[2], Emma L. Lee[3], Namariq Al-Saadi[1], Jeremy Willekens[1,5], Ayush Shah[1], Thushara Nethramangalath[1], Abigail L. Galeano[1], Haiyen Zheng[4], Kalle Gehring[3], Lixia Yue[2], Loren W. Runnels[1,*].

[1]Department of Pharmacology, Rutgers-Robert Wood Johnson Medical School, Piscataway, NJ, USA

[2]Department Cell Biology, Calhoun Cardiology Center, UCONN Health Center, Farmington, CT, USA

[3]Department of Biochemistry, McGill University, Montreal, Canada.

[4]Biological Mass Spectrometry Resources, Robert Wood Johnson Medical School, Rutgers, State University of New Jersey, New Brunswick, NJ, USA

[5]Rutgers Cancer Institute, Rutgers, The State University of New Jersey, New Brunswick, NJ, USA

* Corresponding author email: runnellw@rwjms.rutgers.edu (LR)

Table S1. Description of primers used for the generation of plasmids unique to this publication.

Mutation	Forward primer	Reverse primer
M7-NTERM	AATATGAGAAAAAATTCCTGATATAAGGTCATATTAAGC	GCTTAATATGACCTTATATCAGGAATTTTTTCTCATATT
M7-ST	GAGAGTTCTCCCAGTATACTGAATACAAATTCTGTTCGTCTG	CAGACGAACAGAATTTGTATTCAGTATACTGGGAGAACTCTC
CNNM2-NT	CATCATGAATTC ATG ATT GGC TGT GGC GCT TGT GAA CCC	CATCATAAGCTT G CC CAG GAC GCA GTC CAG CAG TTT GCT A
CNNM2-ΔCNBH	CATCATGAATTCATG ATT GGC TGT GGC GCT TGT GAA CCC	CATCAT AAGCTTCAA GAT TTC AGA TTT GAT GAT TTC TTC AAT CAC ATC TTC C
CNNM2 - T568I	GAAGTTCTGGGAATTGTCATCTTGGAAGATGTGATTGAAG	CTTCAATCACATCTTCCAAGATGACAATTCCCAGAACTTC
CNNM2-S269P	CTGAACATGCCCGGCAGGCACAGCAGC	GCTGCTGTGCCTGCCGGGCATGTTCAG
CNNM2-ΔCBS	GGCTATCCTGGACTTCAACTCTGAAATCTTGGATGAGA	TCTCATCCAAGATTTCAGAGTTGAAGTCCAGGATAGCC
CNNM3-ΔCBS	GTGCTGGACTTCGGCTCCGAGATCCT	GTCCAGGATCTCGGAGCCGAAGTCCA
CNNM2-H523K	CAAGTTTTACAATCACCCTTTGAAGTTCGTTTTCAACGACACCAAGT	ACTTGGTGTCGTTGAAAACGAACTTCAAAGGGTGATTGTAAAACTTG
CNNM2-2XCBS	CATCATGAATTCATG GGC CAG GAG ATA GGC ACG GTC TAT AAC C	CATCAT AAGCTTCAA GAT TTC AGA TTT GAT GAT TTC TTC AAT CAC ATC TTC C
CNBH	CATCATGAATTCATG TTG GAT GAG ACA GAC CTG TAC ACC GAT AAC	CATCATAAGCTTGAT GGC GCC TTC ACT GTG CAG GCT
CNNM2-HA	CAT CAT AAG CTT ATG ATT GGC TGT GGC GCT TGT GAA CCC	CAT CAT GAA TTC GAT GGC GCC TTC ACT GTG CAG GCT G
CNNM2-NT-HA	CAT CAT AAG CTT ATG ATT GGC TGT GGC GCT TGT GAA CCC	CAT CAT GAA TTC G CC CAG GAC GCA GTC CAG CAG TTT GCT A

Table S2: Analysis of Peptide Groups from In Vitro Phosphorylation of CNBH Domain							
**Start**	End	Sequence	Modifications in Master Proteins	Abundance:-KIN	Abundance: +KIN	% Phos.	Site
370	378	ALTASPVPL		33719204	7816922		
717	726	ALTASPVPLS		10746515	3850959		
717	727	ALTASPVPLSL	GST-CNBH 1xPhospho [S726(100)]		37938672		
717	727	ALTASPVPLSL		280193160	79542308		
717	730	ALTASPVPLSLSRT	GST-CNBH 1xPhospho [S726(100)]		3905099.8		
717	731	ALTASPVPLSLSRTF	GST-CNBH 1xPhospho [S726(100)]		159965088		
717	731	ALTASPVPLSLSRTF		26113986	8928077		
717	735	ALTASPVPLSLSRTFVVSR	GST-CNBH 1xPhospho [S726(100)]		9416098		
717	737	ALTASPVPLSLSRTFVVSRTE	GST-CNBH 1xPhospho [S726(98.5)]		6040290.5		
718	727	LTASPVPLSL		14082511	3152871.3	71.5%	S726
718	727	LTASPVPLSL	GST-CNBH 1xPhospho [S726(100)]		6913029.5		
718	731	LTASPVPLSLSRTF	GST-CNBH 1xPhospho [S726(99.2)]		12421988		
719	727	TASPVPLSL	GST-CNBH 1xPhospho [S726(100)]		77667956		
719	727	TASPVPLSL		156930552	39286771		
719	728	TASPVPLSLS		2377075.8	913129.5		
719	730	TASPVPLSLSRT	GST-CNBH 1xPhospho [S726(100)]		2037293.4		
720	727	ASPVPLSL	GST-CNBH 1xPhospho [S726(100)]		74328654		
720	727	ASPVPLSL		55954000	12553178		
761	787	SLSRSDRIDAMTPTLGSSNNQL SSSFL		31809390	50390016		
761	787	SLSRSDRIDAMTPTLGSSNNQL SSSFL	S783/S784 (manual)		4232620.5		
761	787	SLSRSDRIDAMTPTLGSSNNQL SSSFL		4624258.5	6520434.5		
761	797	SLSRSDRIDAMTPTLGSSNNQL SSSFLQVYIPDYSVR		5329698.5	7928186.5		
761	797	SLSRSDRIDAMTPTLGSSNNQL SSSFLQVYIPDYSVR	S783/S784 (manual)		2527291.5		
761	801	SLSRSDRIDAMTPTLGSSNNQL SSSFLQVYIPDYSVRALSD		519207.91	2265305.5		
765	786	SDRIDAMTPTLGSSNNQLSSSF		9879601.5	13162851		
768	786	IDAMTPTLGSSNNQLSSSF		9250169	13544671		
768	786	IDAMTPTLGSSNNQLSSSF		2583396.3	2352852.8		
770	786	AMTPTLGSSNNQLSSSF		837722724	844115324		
770	786	AMTPTLGSSNNQLSSSF		19463782	21468476		
770	787	AMTPTLGSSNNQLSSSFL		201568624	206193424		
770	787	AMTPTLGSSNNQLSSSFL			41309088		
770	788	AMTPTLGSSNNQLSSSFLQ		4517285	4822970		
423	789	AMTPTLGSSNNQLSSSFLQV	GST-CNBH 1xPhospho [S783(98.7)]		7049230		
770	797	AMTPTLGSSNNQLSSSFLQVYI PDYSVR		3330274.8	4229313		
770	779	AMTPTLGSSNNQLSSSFLQVYI PDYSVR			1678337.1	2.16%	S783/ 784
771	786	MTPTLGSSNNQLSSSF		60717308	64569664		
771	787	MTPTLGSSNNQLSSSFL		14672938	16150741		
771	787	MTPTLGSSNNQLSSSFL			3121074.5		
772	782	TPTLGSSNNQL		3009893.5	4248382.5		
772	786	TPTLGSSNNQLSSSF		13276192	15689118		
772	787	TPTLGSSNNQLSSSFL		86980688	84711184		
772	787	TPTLGSSNNQLSSSFL	GST-CNBH 1xPhospho [S783(99.6)]		17359078		
772	797	TPTLGSSNNQLSSSFLQVYIPDY SVR		2199618	3187141.3		
772	797	TPTLGSSNNQLSSSFLQVYIPDY SVR			767416.94		

Table S2: Analysis of Peptide Groups from In Vitro Phosphorylation of CNBH Domain							
**Start**	End	Sequence	Modifications in Master Proteins	Abundance:-KIN	Abundance: +KIN	% Phos.	Site
**804**	823	FVKISRQQYQNALMASRMDK		17141580	22674908		
**804**	834	FVKISRQQYQNALMASRMDKTPQSSDSENTK		2949777.3	4678597		
**804**	836	FVKISRQQYQNALMASRMDKTPQSSDSENTKIE		62502717	115544928		
**804**	837	FVKISRQQYQNALMASRMDKTPQSSDSENTKIEL		19962195	33583683		
**804**	839	FVKISRQQYQNALMASRMDKTPQSSDSENTKIELTL		28328636	49654952		
**805**	823	VKISRQQYQNALMASRMDK		6606817.5	7829505.5		
**805**	834	VKISRQQYQNALMASRMDKTPQSSDSENTK		2496796.5	3624328.8		
**805**	836	VKISRQQYQNALMASRMDKTPQSSDSENTKIE		12529757	18410224		
**805**	839	VKISRQQYQNALMASRMDKTPQSSDSENTKIELTL		19592593	24197180		
**807**	823	ISRQQYQNALMASRMDK		43810108	57788920	1.27%	S819
**807**	834	ISRQQYQNALMASRMDKTPQSSDSENTK		10770178	17079726		
**807**	836	ISRQQYQNALMASRMDKTPQSSDSENTKIE		5388258	9943944		
**807**	836	ISRQQYQNALMASRMDKTPQSSDSENTKIE		3147947.8	4396676		
**807**	836	ISRQQYQNALMASRMDKTPQSSDSENTKIE	GST-CNBH 1xPhospho [S819(99.5)]		5557553.5		
**807**	837	ISRQQYQNALMASRMDKTPQSSDSENTKIEL		4244775	7907957		
**807**	837	ISRQQYQNALMASRMDKTPQSSDSENTKIELTL		26298177	44073063		
**810**	823	QQYQNALMASRMDK		1692174.9	3115156.5		
**810**	836	QQYQNALMASRMDKTPQSSDSENTKIE		3736047.3	5313930.5		
**818**	836	ASRMDKTPQSSDSENTKIE		3695740.3	3206799.8		

[1]Percent phosphorylation was calculated using summed abundance of all phosphorylated peptides divided by abundance of all peptides covering that site regardless of modification status.

Figure S1 – Source data from Figures 1, 2, 3, and Figure S3.

Uncropped blots that were used in Figures 1, 2, 3, and Figure S3.

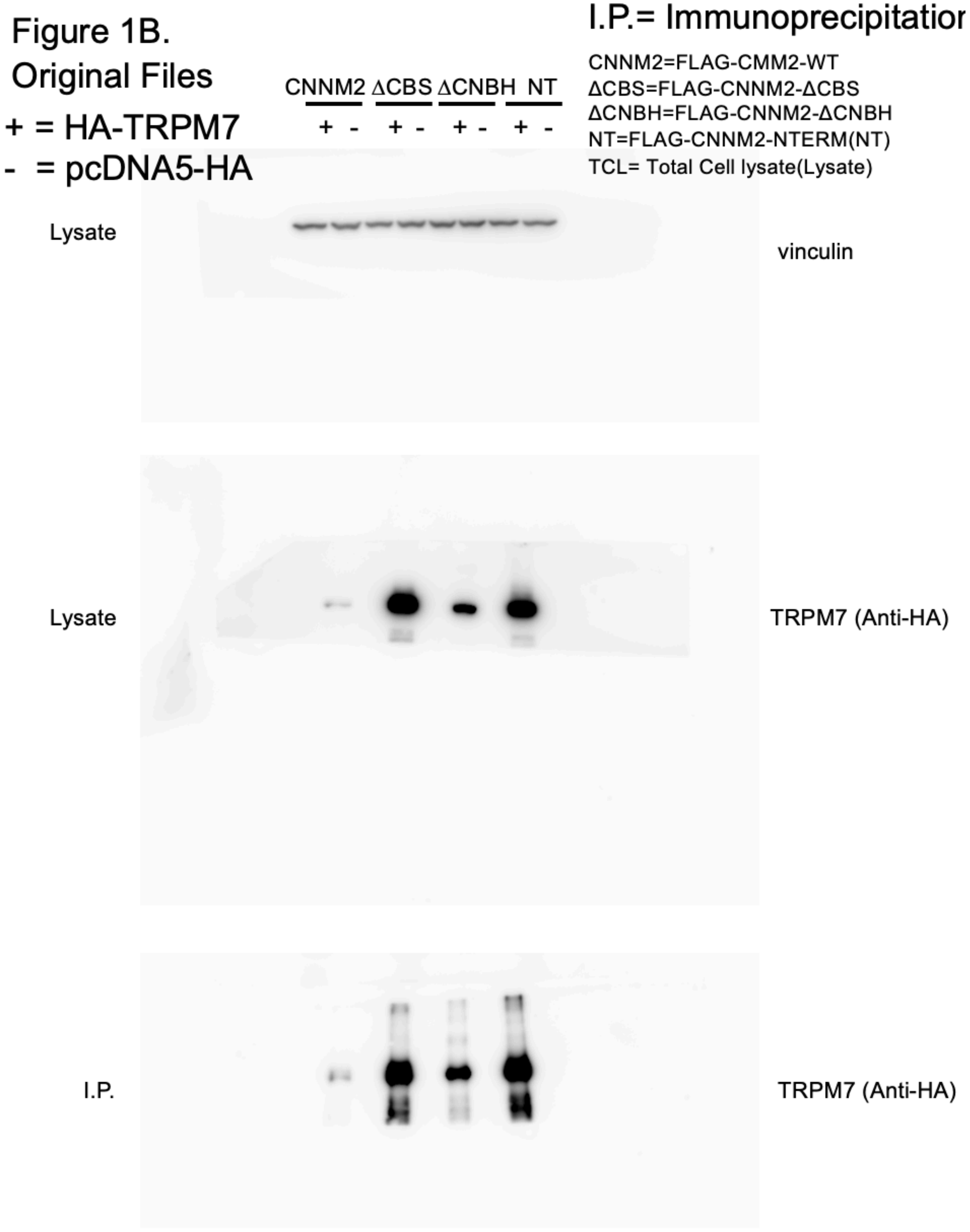

Figure S1 – Source data from Figures 1, 2, 3, and Figure S3.

Uncropped blots that were used in Figures 1, 2, 3, and Figure S3.

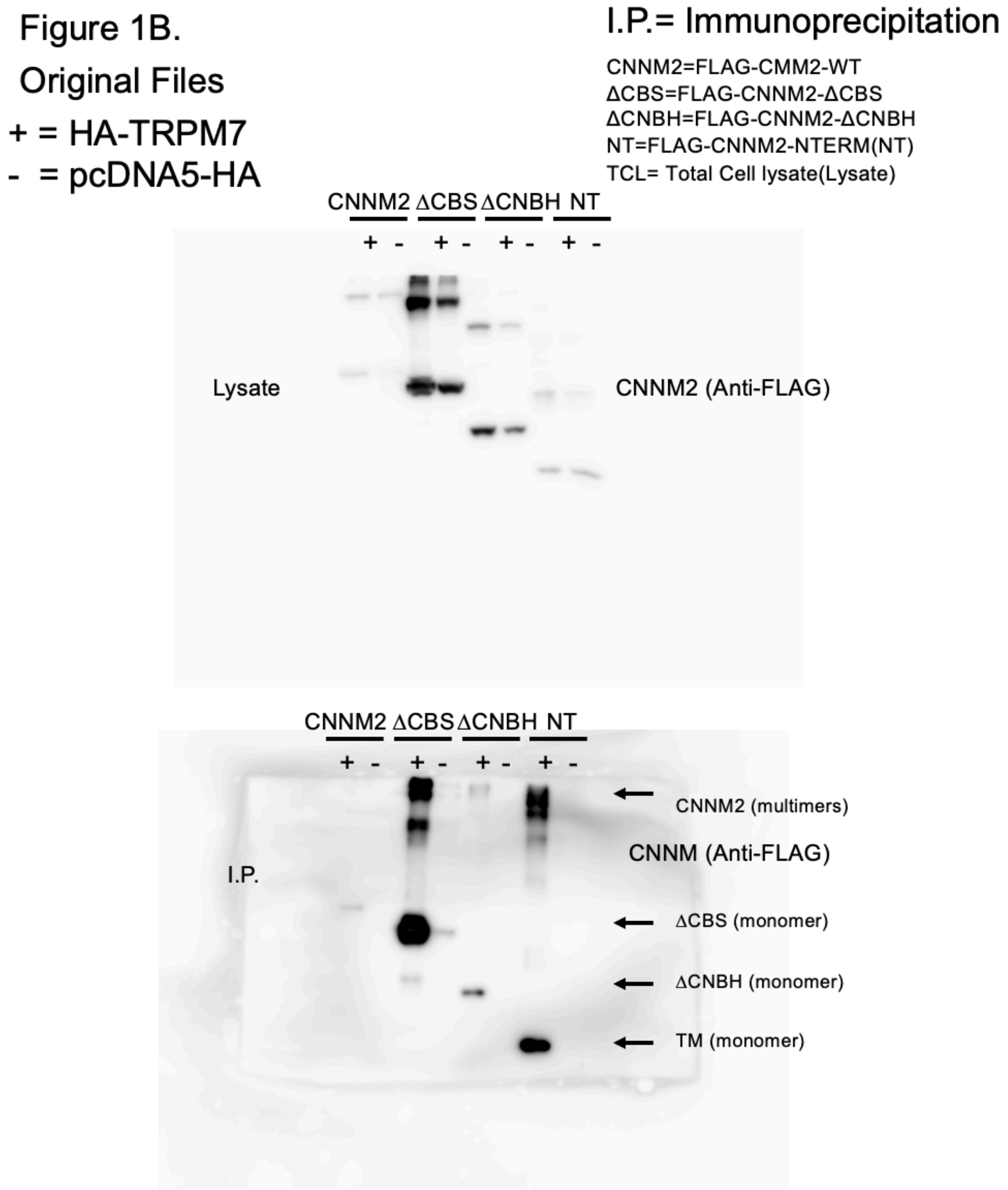

Figure S1 – Source data from Figures 1, 2, 3, and Figure S3.

Uncropped blots that were used in Figures 1, 2, 3, and Figure S3.

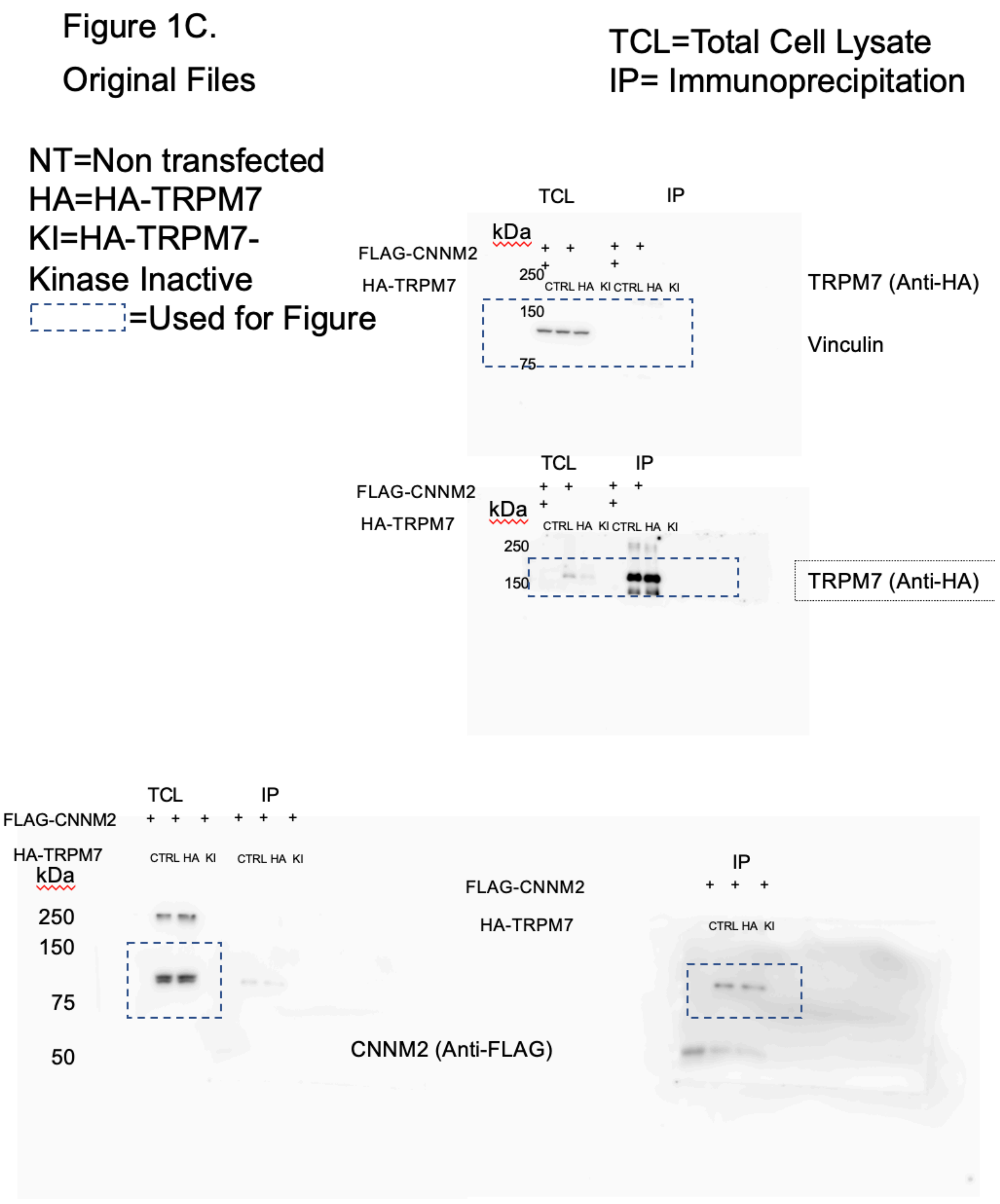

Figure S1 – Source data from Figures 1, 2, 3, and Figure S3.

Uncropped blots that were used in Figures 1, 2, 3, and Figure S3.

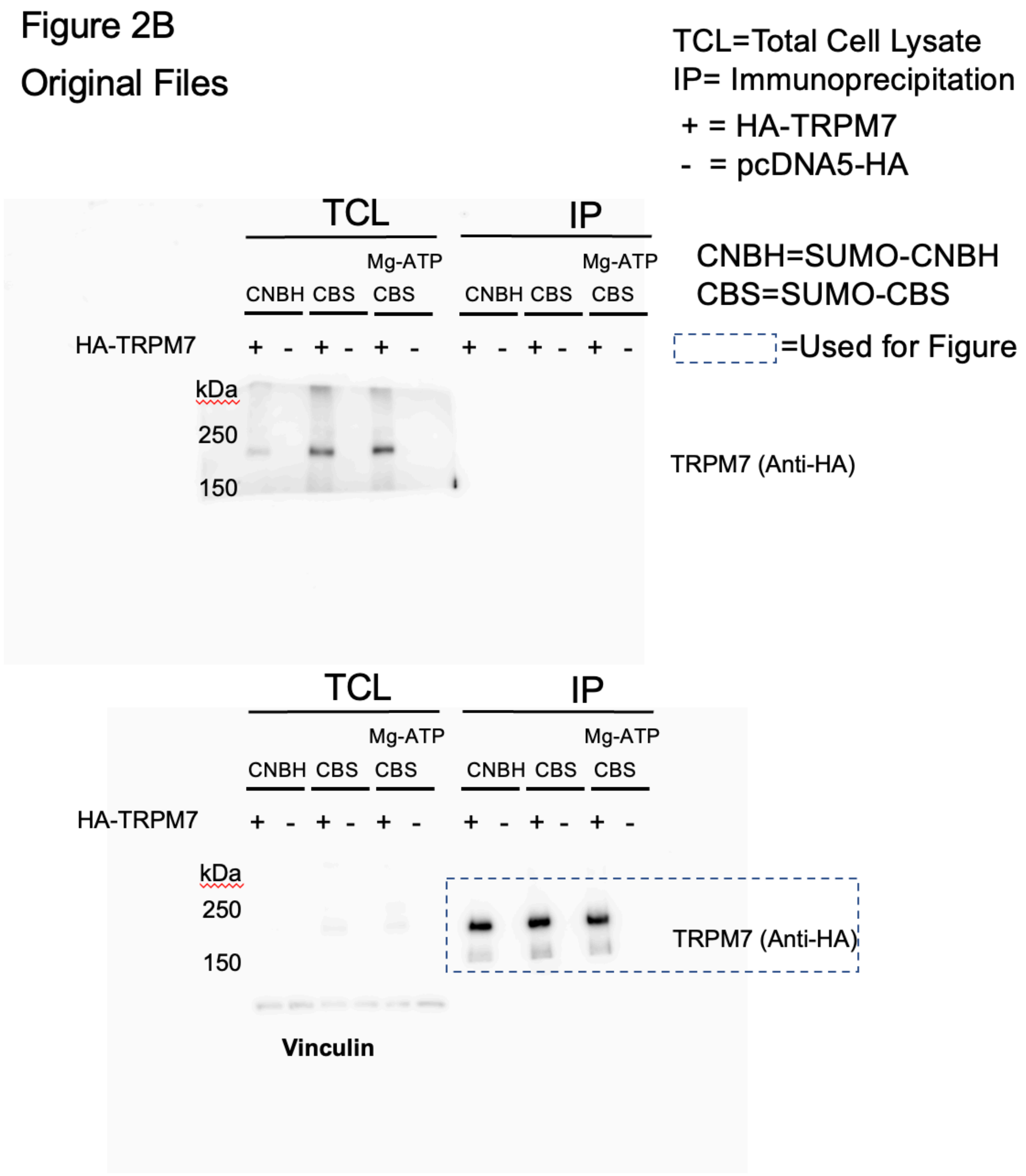

Figure S1 – Source data from Figures 1, 2, 3, and Figure S3.

Uncropped blots that were used in Figures 1, 2, 3, and Figure S3.

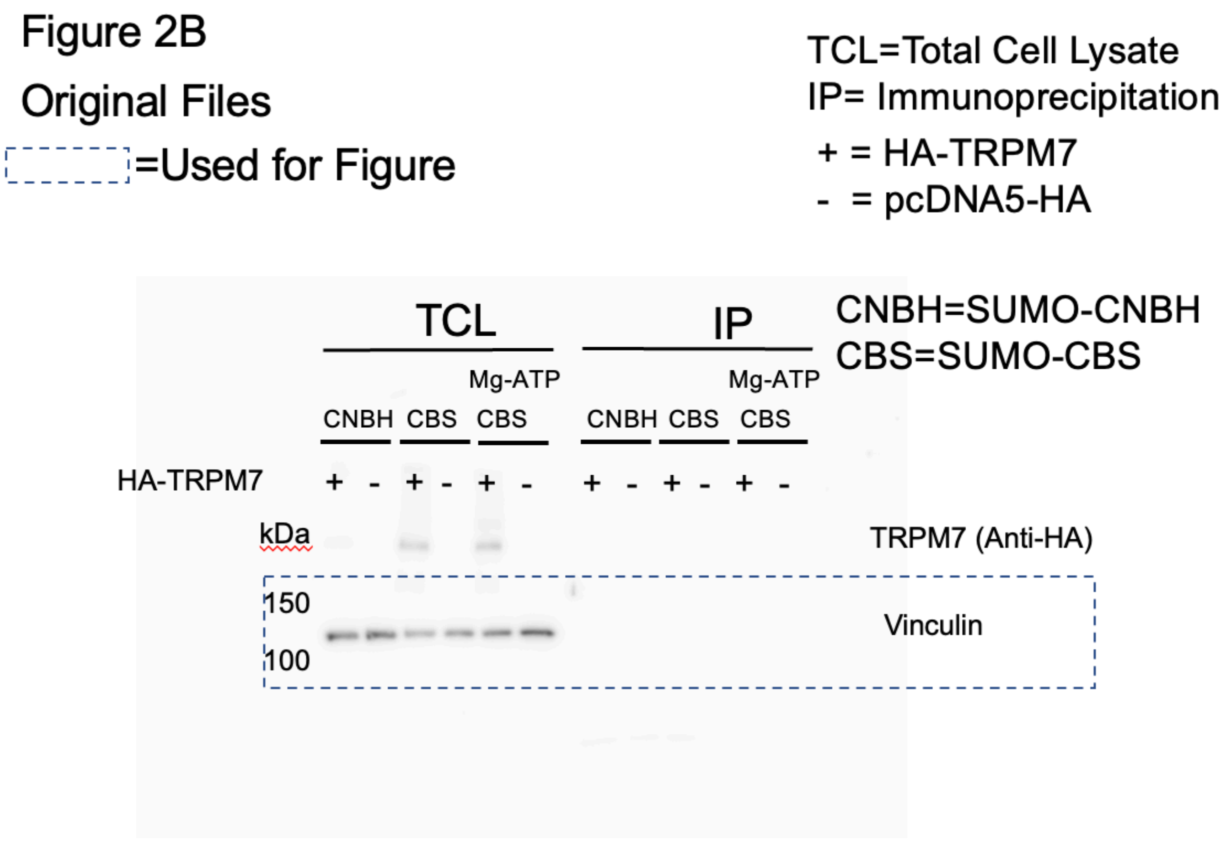

Figure S1 – Source data from Figures 1, 2, 3, and Figure S3.

Uncropped blots that were used in Figures 1, 2, 3, and Figure S3.

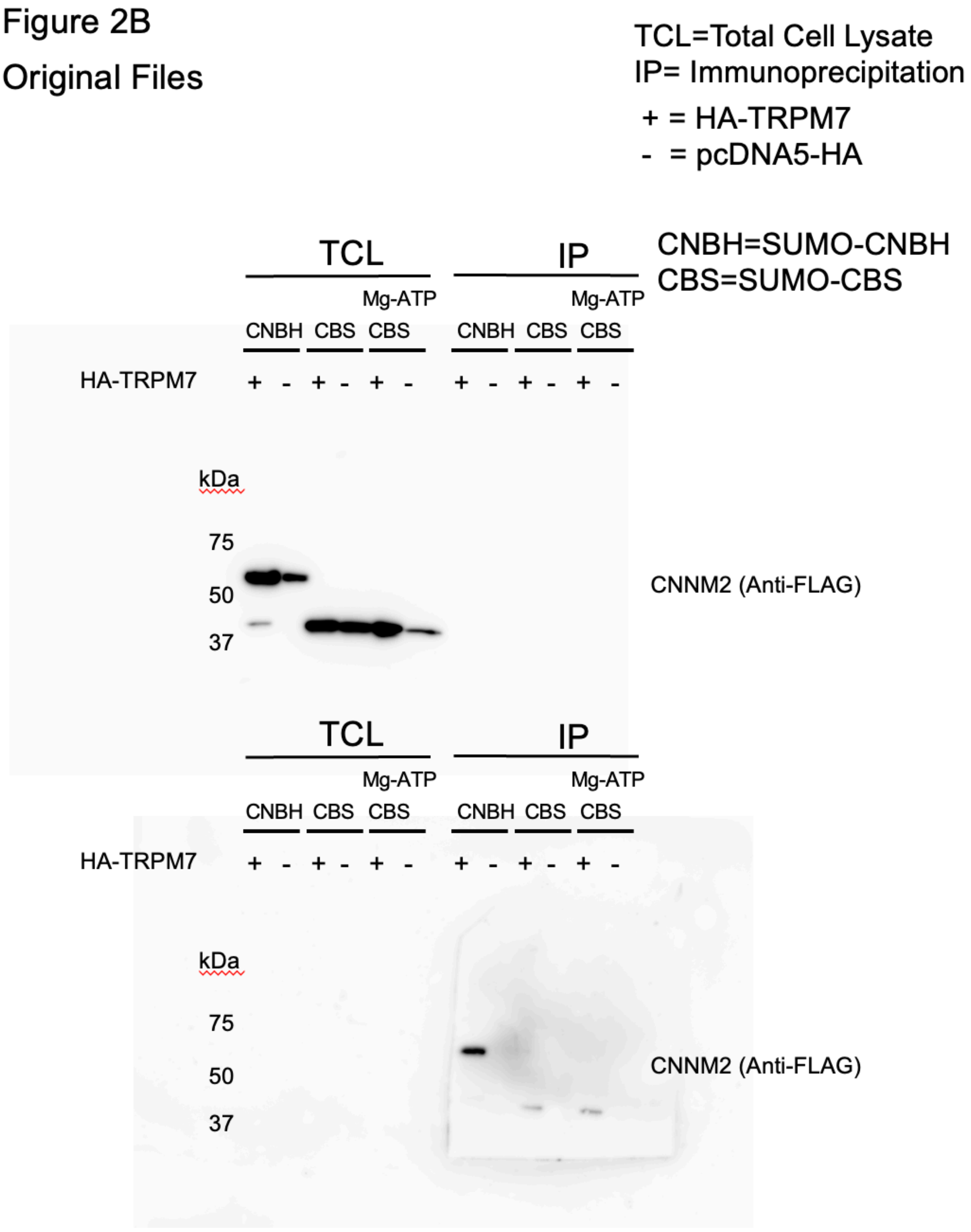

Figure S1 – Source data from Figures 1, 2, 3, and Figure S3.

Uncropped blots that were used in Figures 1, 2, 3, and Figure S3.

Figure 3B

Original Files

M7-NTERM=HA-TRMP7-NTERM
M7-CTERM=mfGFP-TRPM7-CTERM

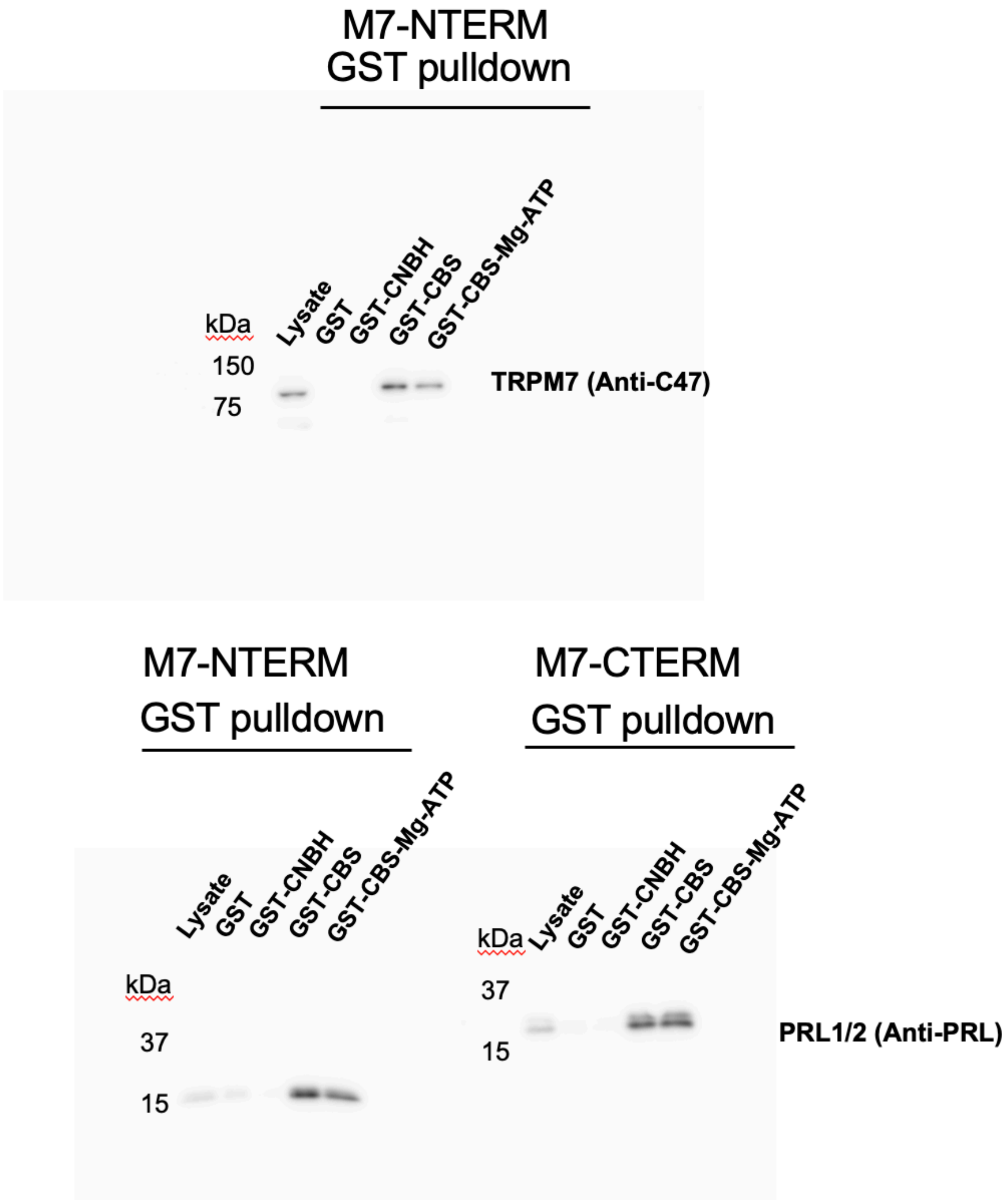

Figure S1 – Source data from Figures 1, 2, 3, and Figure S3.

Uncropped blots that were used in Figures 1, 2, 3, and Figure S3.

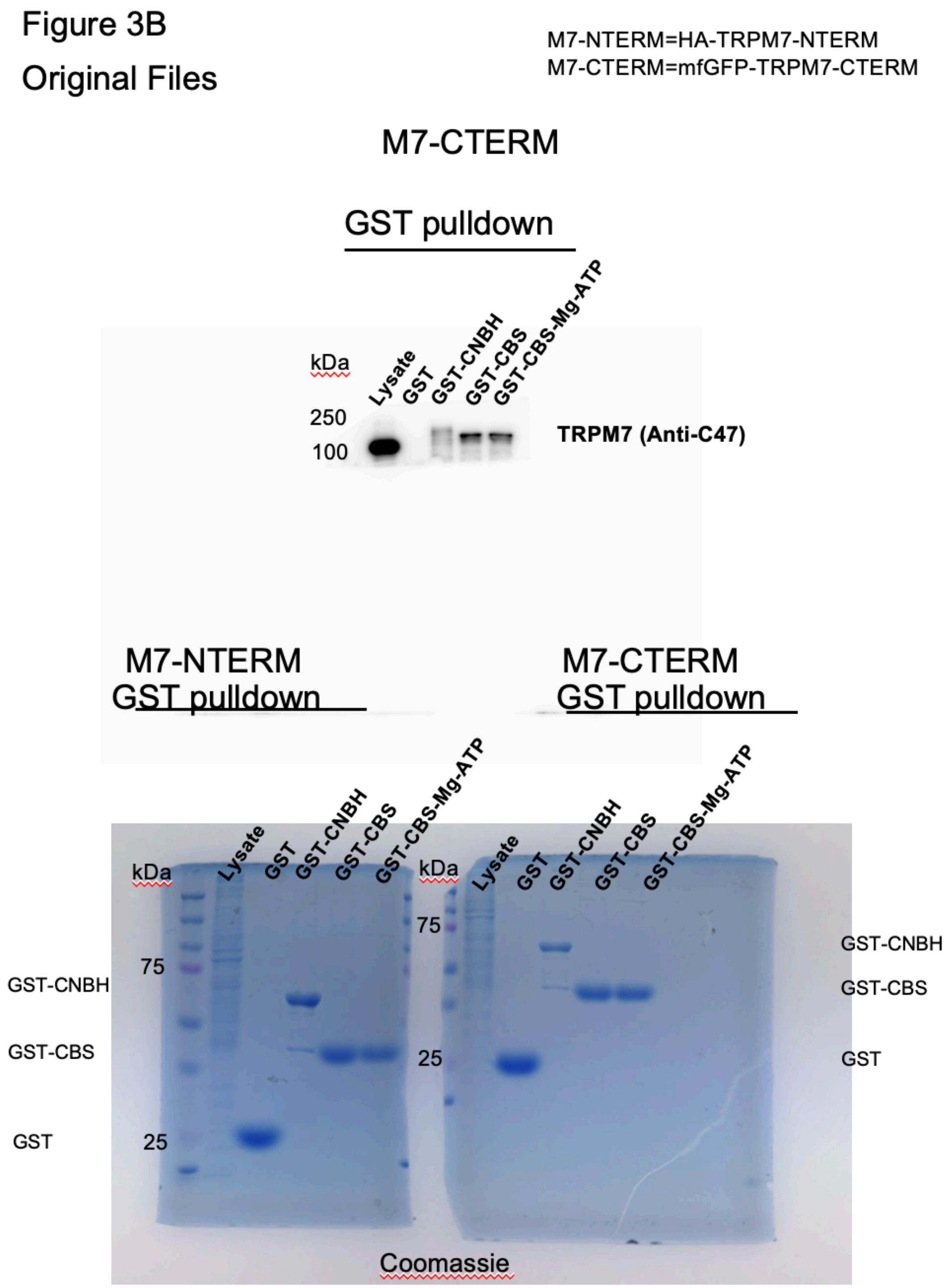

Figure S1 – Source data from Figures 1, 2, 3, and Figure S3.

Uncropped blots that were used in Figures 1, 2, 3, and Figure S3.

Figure 3C
Original Files

M7-CTERM=mfGFP-TRPM7-CTERM
M7-CTERM-KI=mfGFP-TRPM7-CTERM-KI

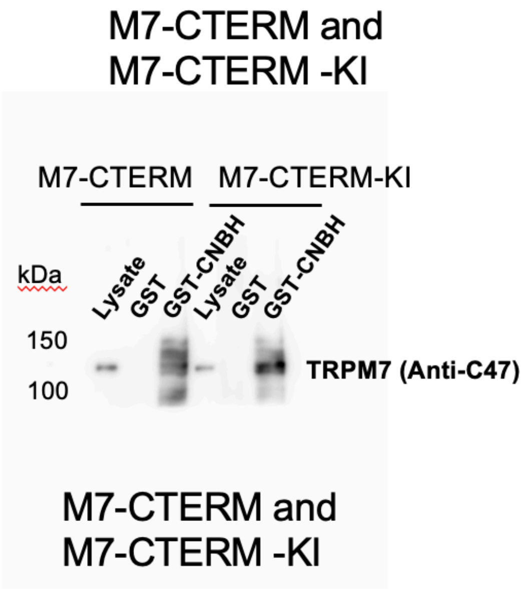

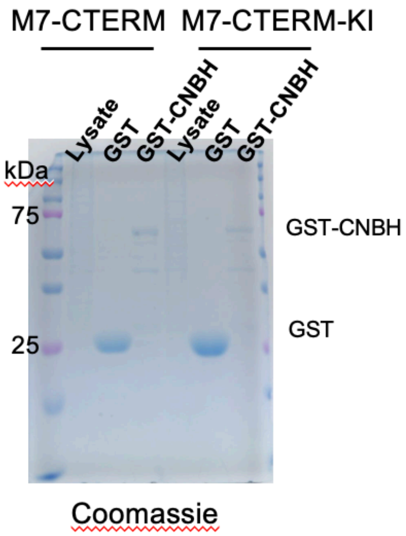

Figure S1 – Source data from Figures 1, 2, 3, and Figure S3.

Uncropped blots that were used in Figures 1, 2, 3, and Figure S3.

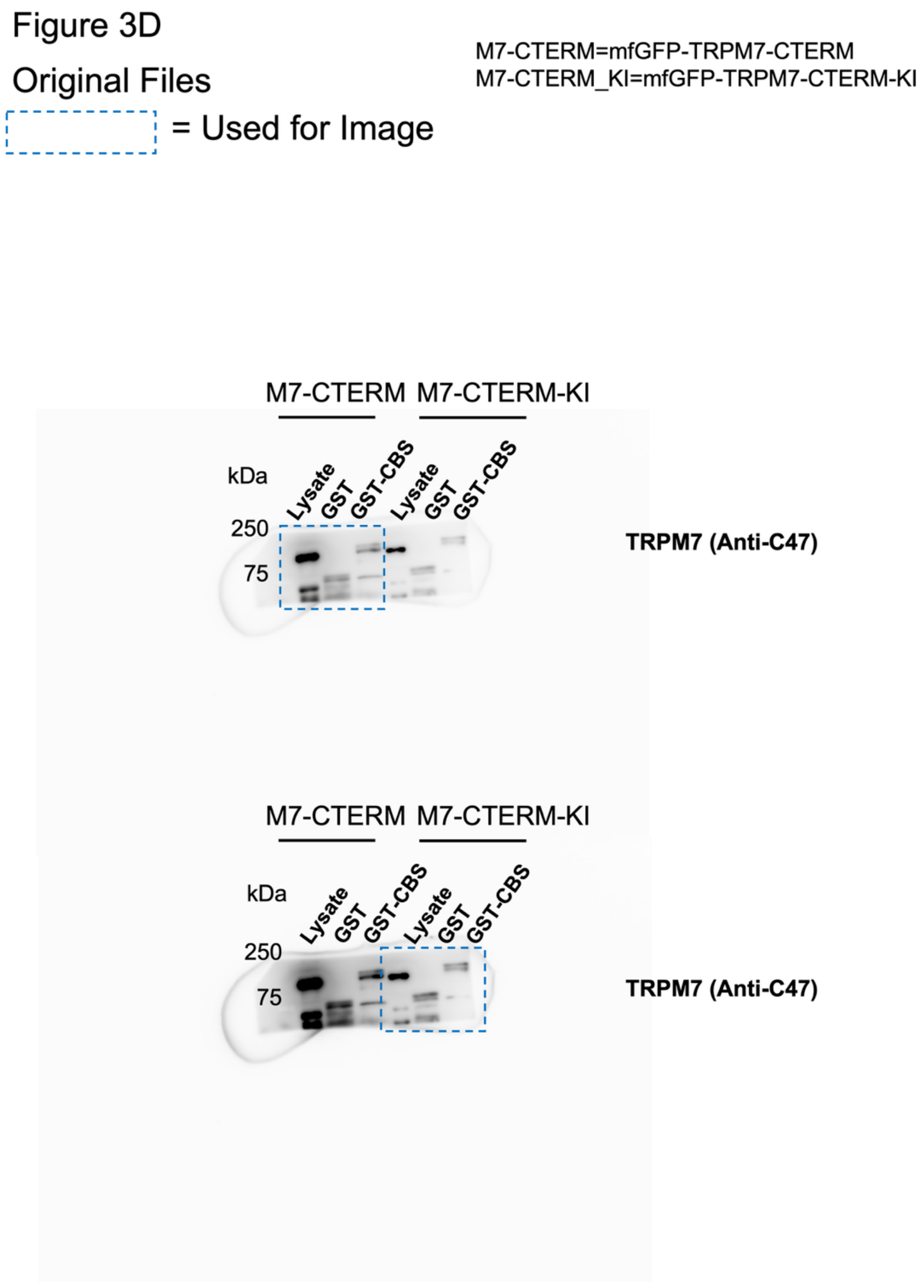

Figure S1 – Source data from Figures 1, 2, 3, and Figure S3.

Uncropped blots that were used in Figures 1, 2, 3, and Figure S3.

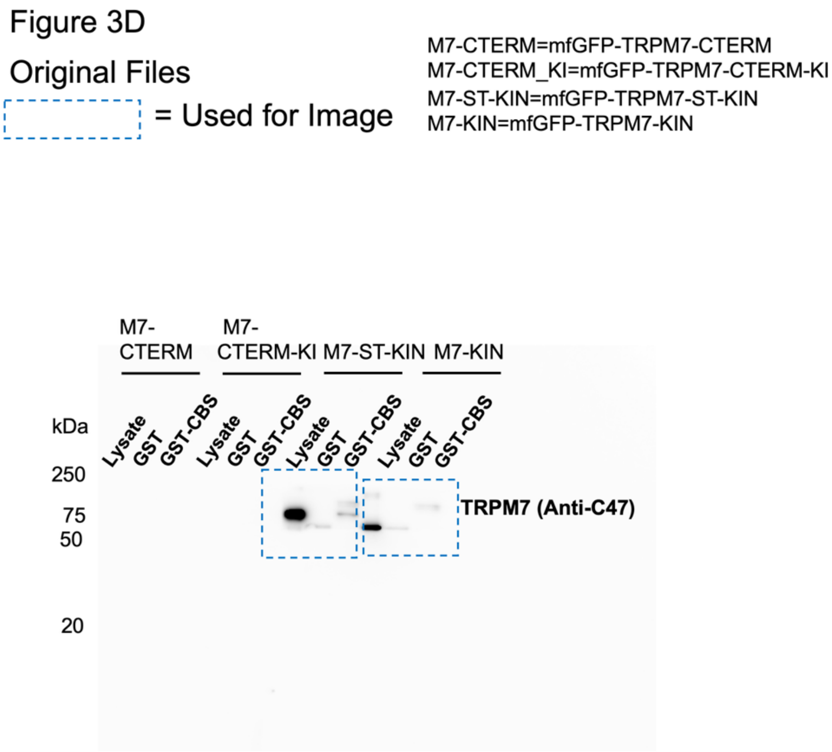

Figure S1 – Source data from Figures 1, 2, 3, and Figure S3.

Uncropped blots that were used in Figures 1, 2, 3, and Figure S3.

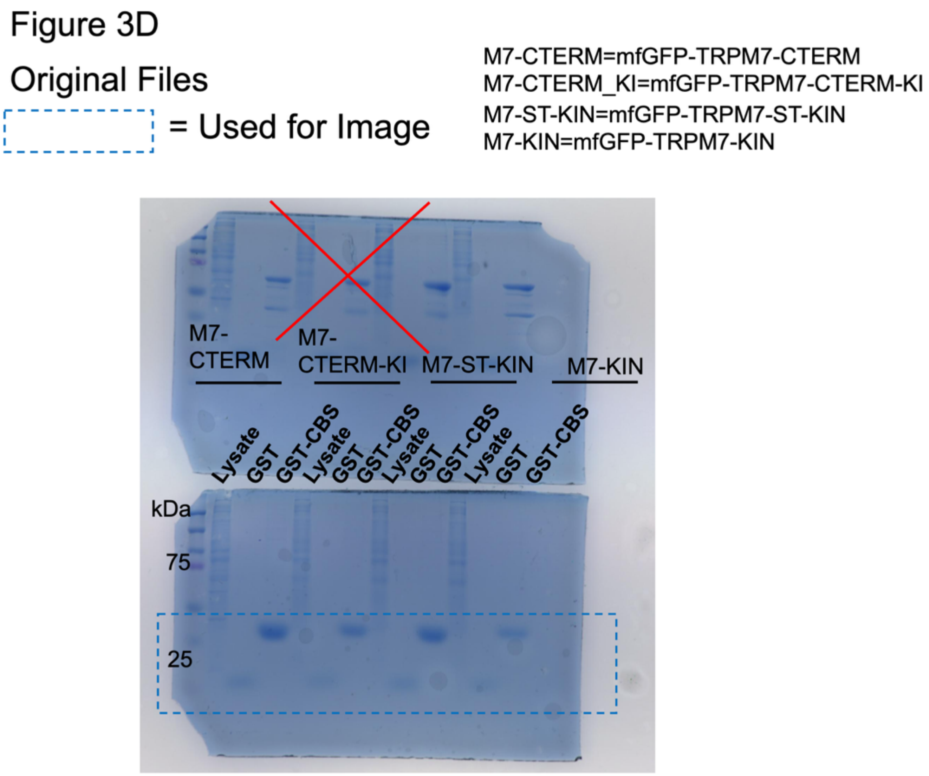

Figure S1 – Source data from Figures 1, 2, 3, and Figure S3.

Uncropped blots that were used in Figures 1, 2, 3, and Figure S3.

Figure 3E

Original Files

M7-ST-KIN=mfGFP-TRPM7-ST-KIN
M7-KIN=mfGFP-TRPM7-KIN

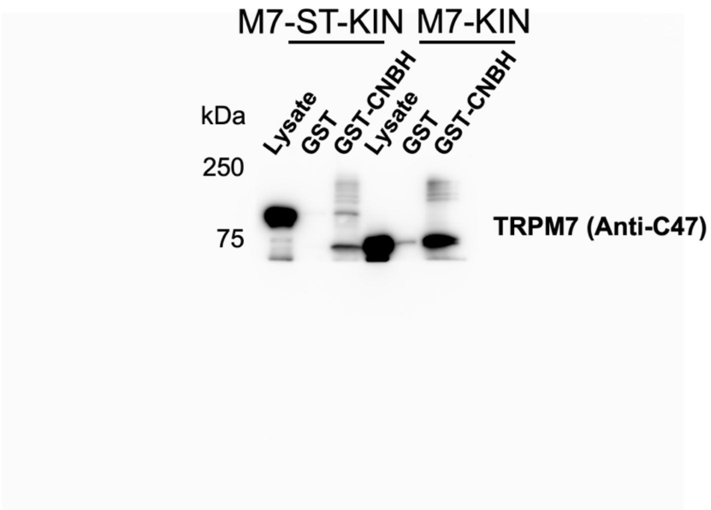

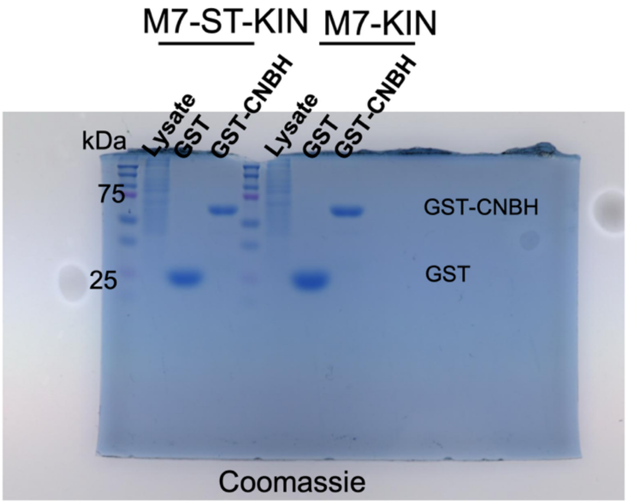

Figure S1 – Source data from Figures 1, 2, 3, and Figure S3.

Uncropped blots that were used in Figures 1, 2, 3, and Figure S3.

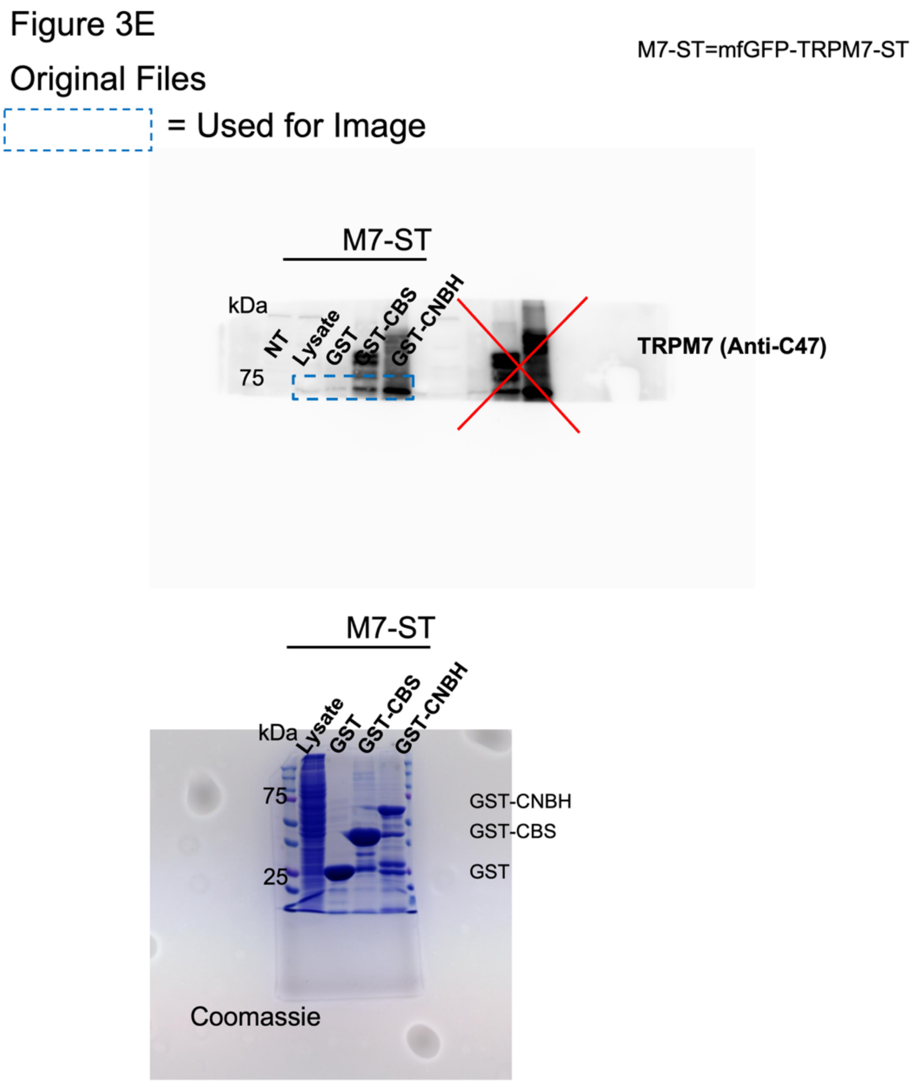

Figure S1 – Source data from Figures 1, 2, 3, and Figure S3.

Uncropped blots that were used in Figures 1, 2, 3, and Figure S3.

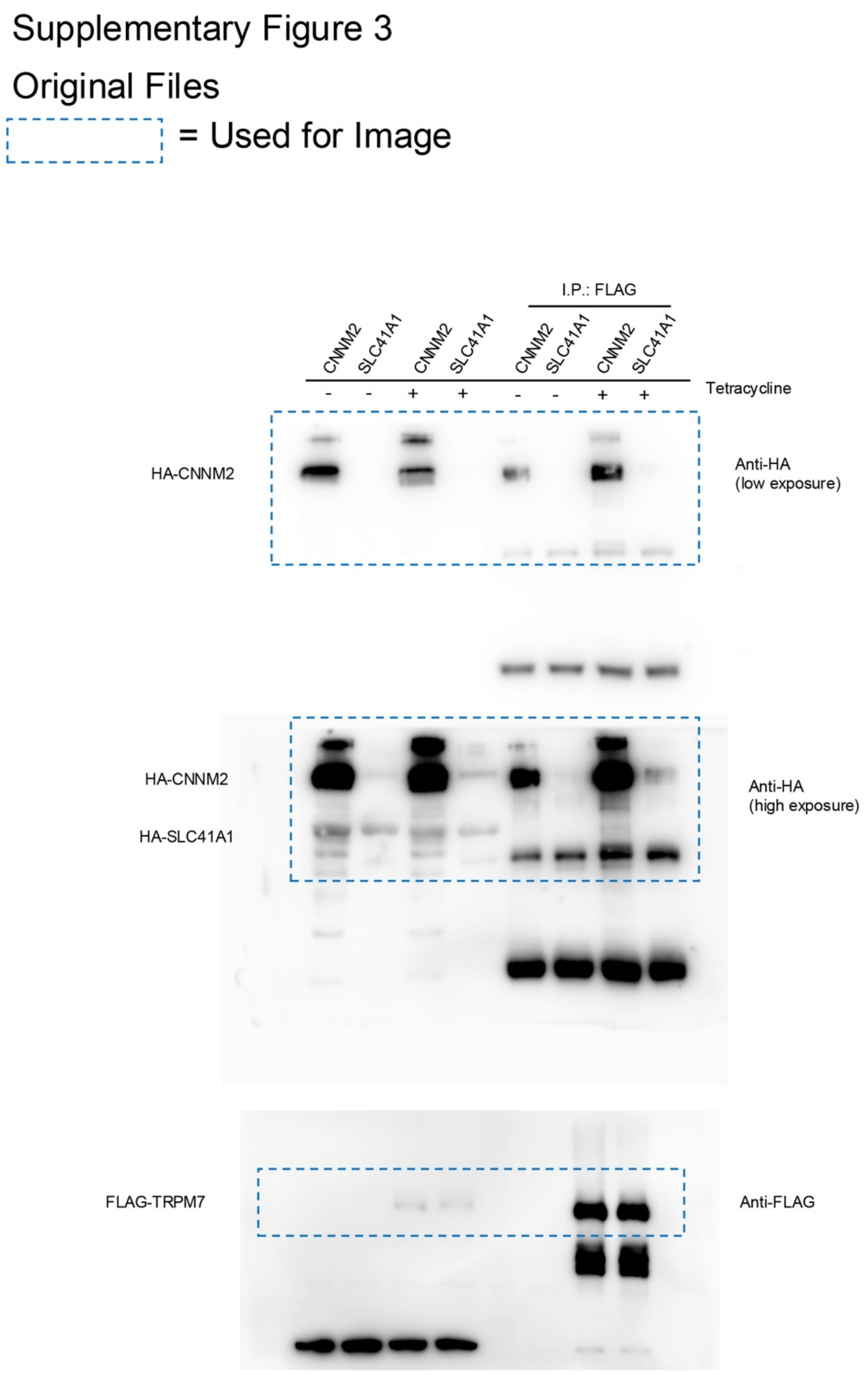

Figure S1 – Source data from Figures 1, 2, 3, and Figure S3.

Uncropped blots that were used in Figures 1, 2, 3, and Figure S3.

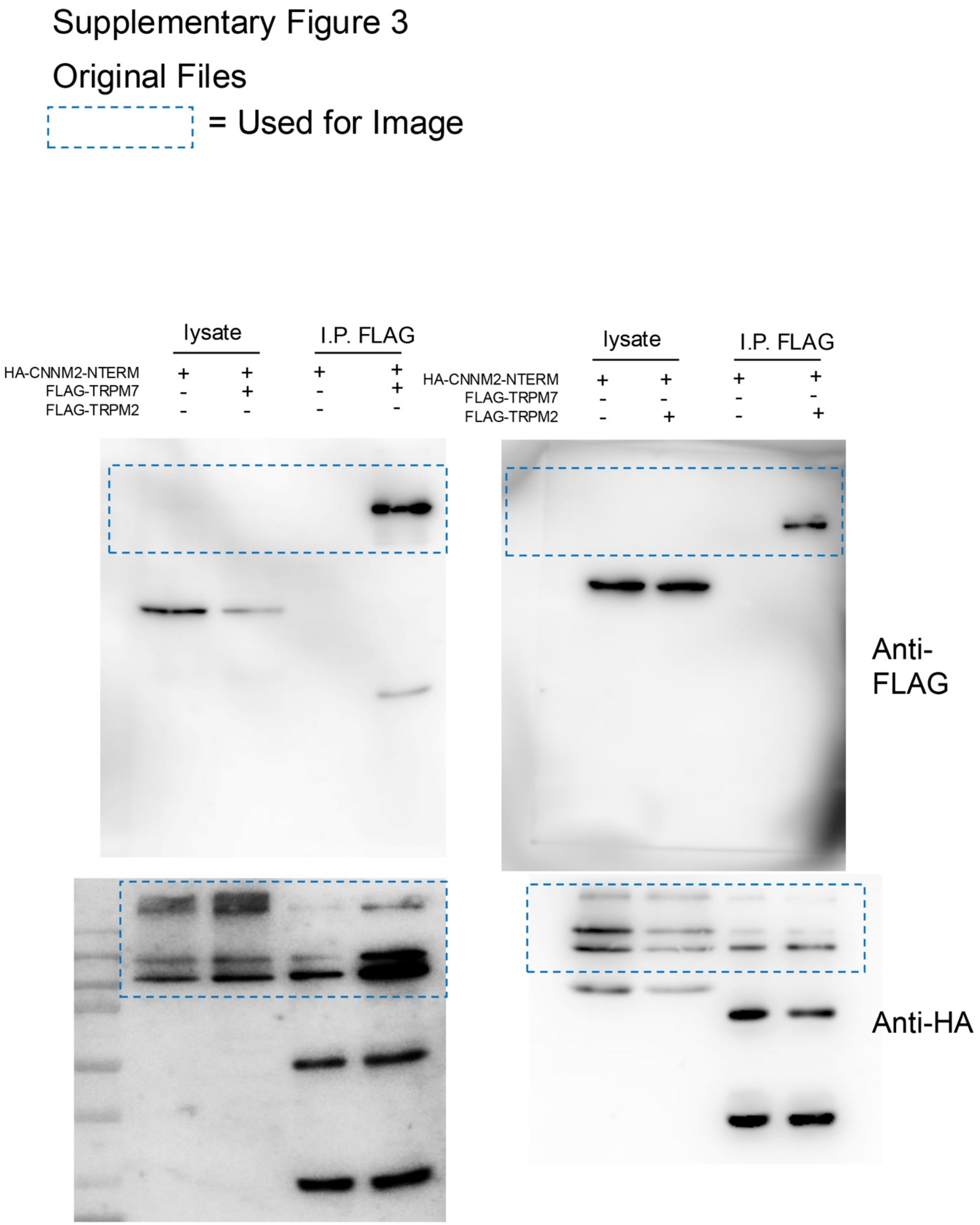

Figure S2 – Replicate experiments from Figure 1. See Figure 1 legend.

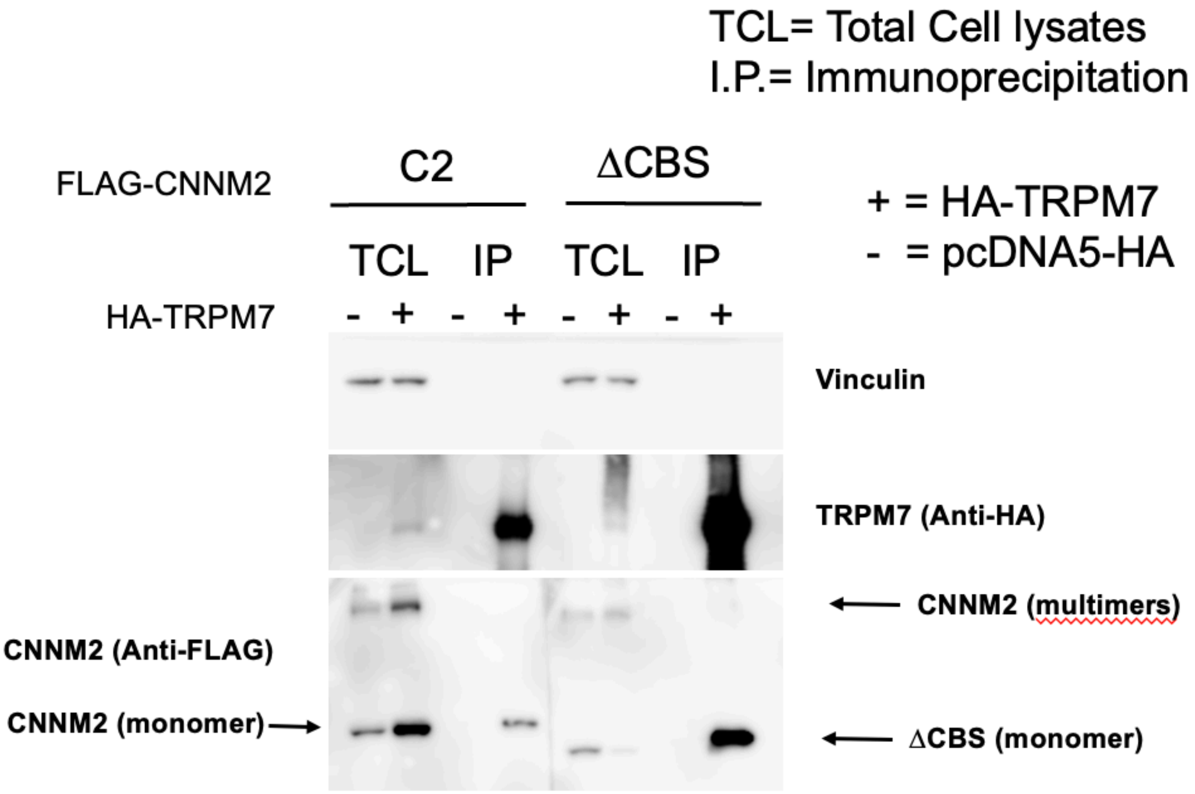

Figure 1B Replicate (CNNM2 & CNNM2ΔCBS)

Figure S2 – Replicate experiments from Figure 1. See Figure 1 legend.

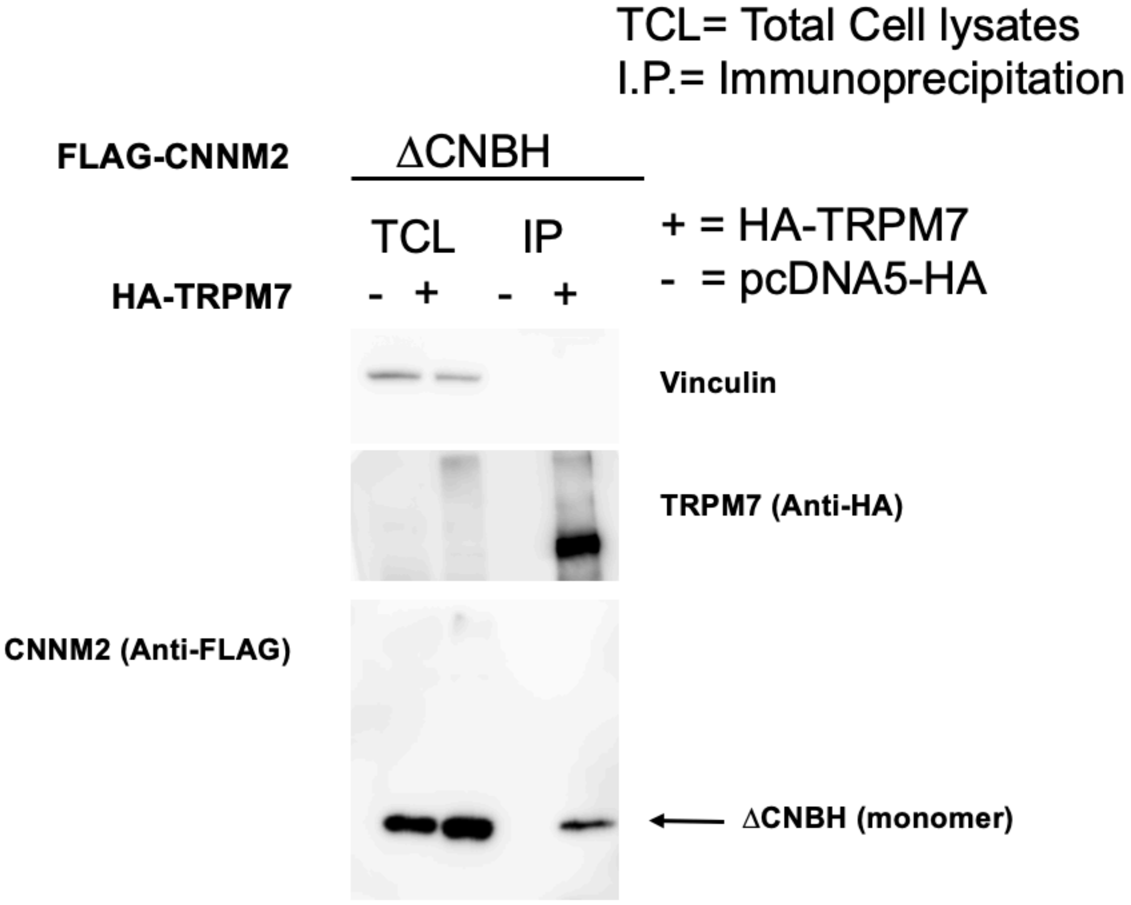

Figure 1B Replicate (CNNM2ΔCNBH)

Figure S2 – Replicate experiments from Figure 1. See Figure 1 legend.

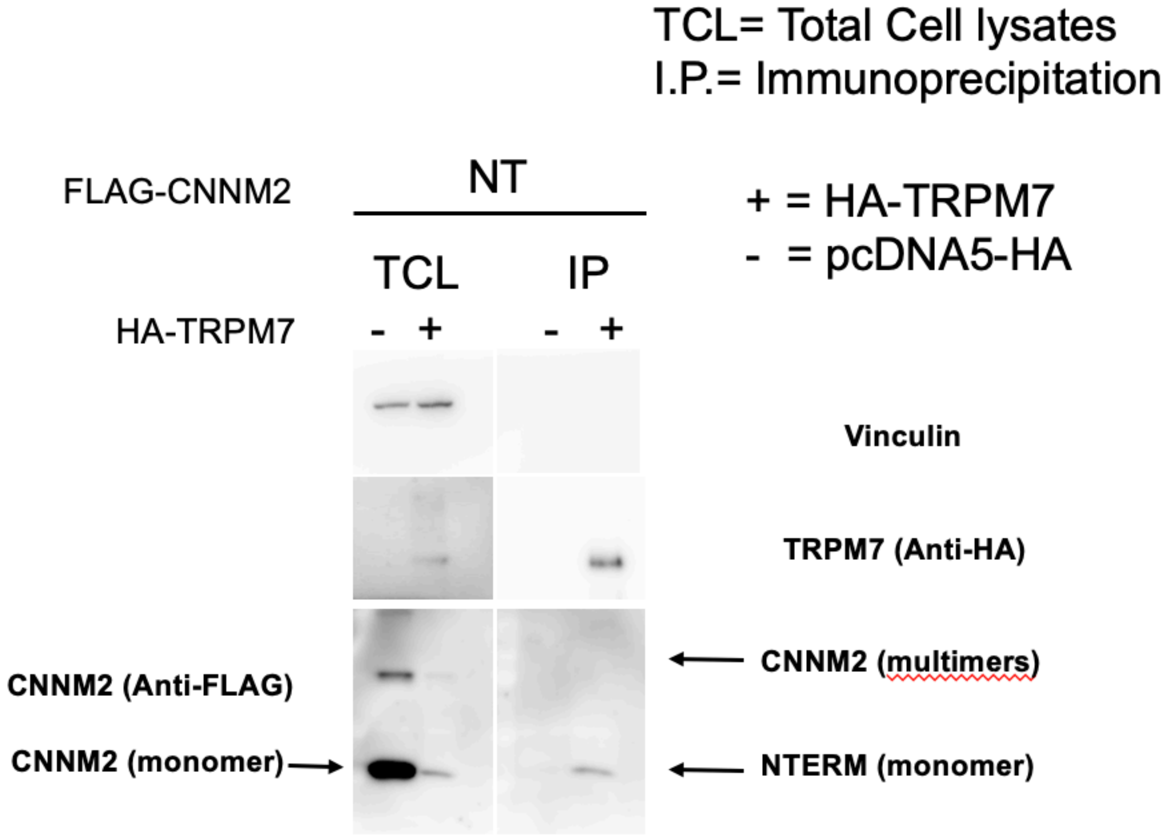

Figure 1B Replicate (CNNM2-NTERM (NT))

Figure S3 – TRPM7 interacts specifically with CNNM2.

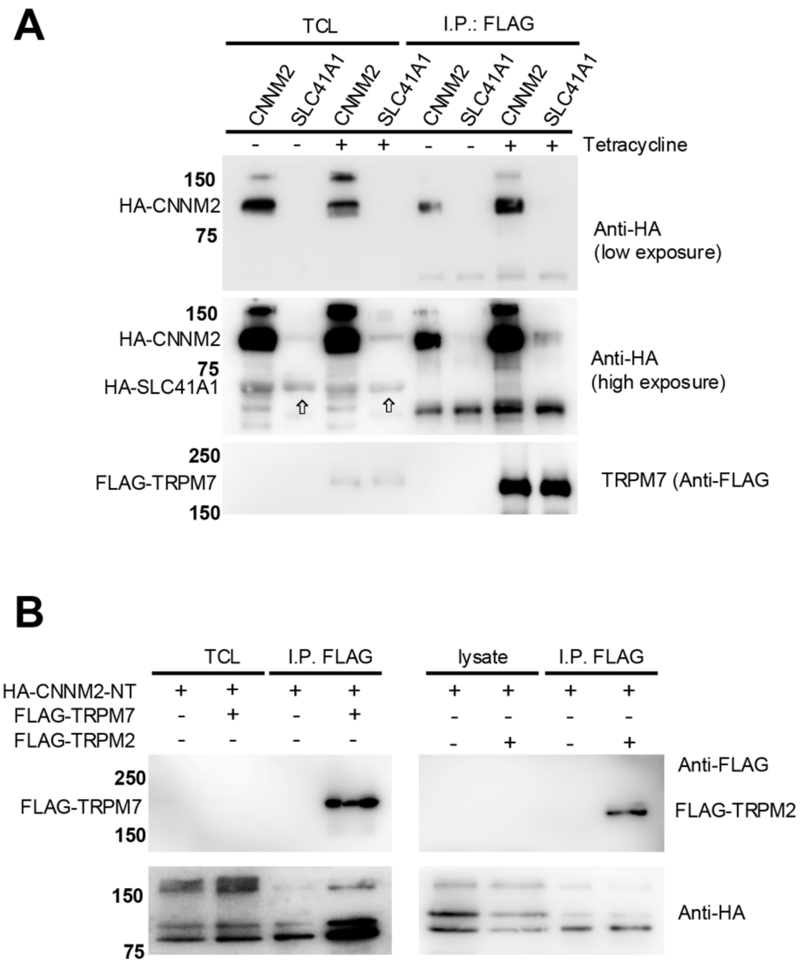

Figure S3. TRPM7 interacts specifically with CNNM2.

(A) HA-tagged CNNM2 (HA-CNNM2) or SLC41A1 (HA-SLC41A1) were co-expressed with FLAG-tagged TRPM7 (FLAG-TRPM7) as indicated. CNNM2 migrates in the SDS-PAGE gel as a monomer (~90-100 kDa) and higher order oligomer (~150 kDa). SLC41A1 migrates in the SDA-PAGE gel as a monomer (~50 kDa), as indicated by arrows on the blot. Cells were lysed, and TRPM7 was immunoprecipitated from lysate using Pierce™ Anti-DYKDDDDK magnetic agarose. CNNM2 co-immunoprecipitated with TRPM7, whereas SLC41A1 did not. **(B)** HA-tagged CNNM2 (HA-CNNM2) was co-expressed with FLAG-TRPM7 or FLAG-tagged TRPM2 (FLAG-TRPM2) as indicated. Following lysis, TRPM7 and TRPM2 was immunoprecipitated from lysate using Pierce™ Anti-DYKDDDDK magnetic agarose. HA-CNNM2 specifically co-immunoprecipitated with FLAG-TRPM7 but not FLAG-TRPM2.

Figure S4 – The CNNM2 N-terminal domain is primarily localized intracellularly in 293-TRPM7 cells.

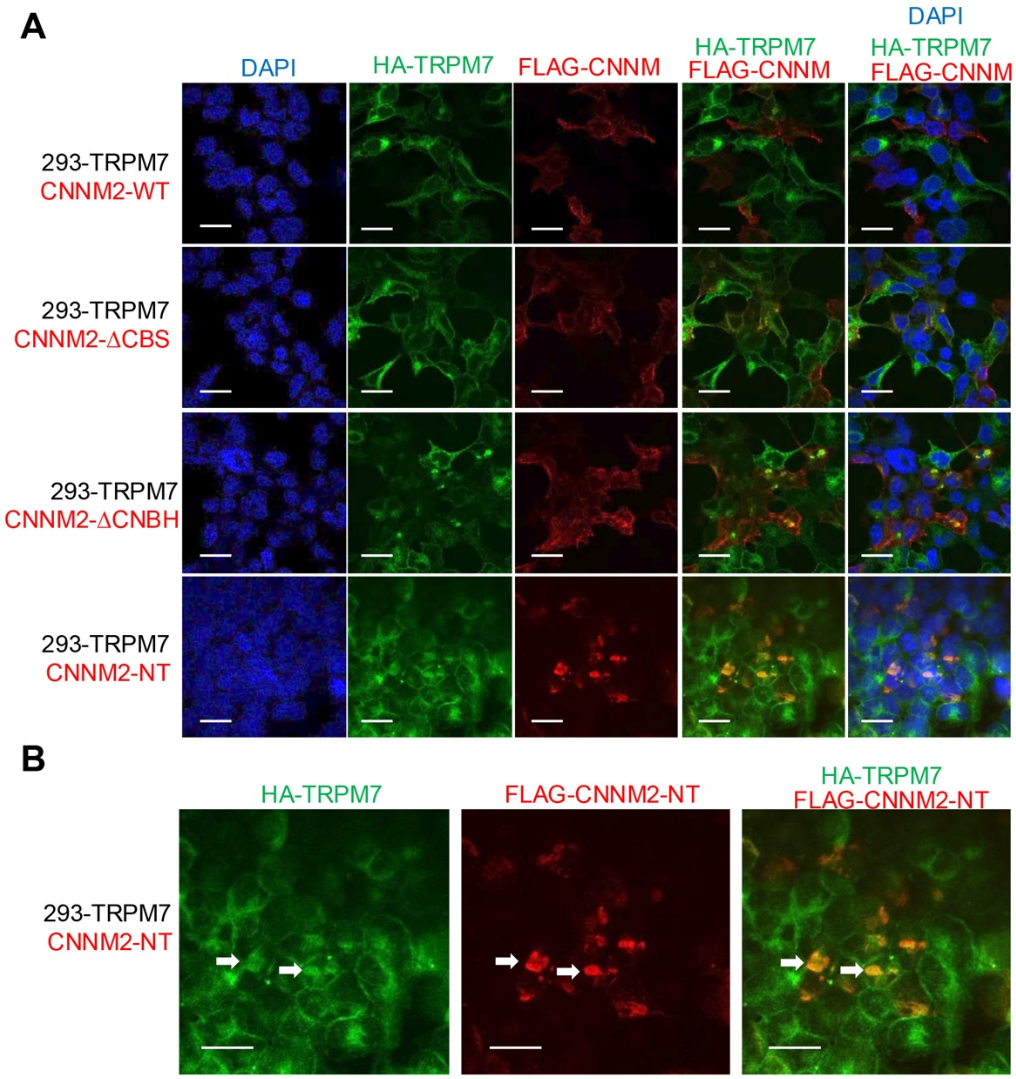

Figure S4. The CNNM2 N-terminal domain is primarily localized intracellularly in 293-TRPM7 cells. (A) 293-TRPM7 cells, which express HA-tagged TRPM7 in response to tetracycline, were transfected with FLAG-tagged CNNM2 constructs: wild-type (CNNM2-WT), CNNM2-ΔCBS, CNNM2-ΔCNBH, and CNNM2-NT, to assess the cellular localization of the proteins by immunocytochemistry and fluorescence microscopy. CNNM2-WT, CNNM2-ΔCBS, and CNNM2-ΔCNBH were detected at the cell periphery. CNNM2-NT, which lacks both the CBS and CNBH domain, had an altered localization compared to the other CNNM2 constructs. In contrast, CNNM2-NT, which lacks the CBS pair and CNBH domains, showed

altered localization and was predominantly intracellularly, accumulating in puncta that co-localized with TRPM7. White scale bars represent 10 microns. **(B)** Enlarged images of CNNM2-NT and TRPM7 from (A), with white arrows indicate intracellular CNNM2-N2-positive puncta.

Figure S5 – Replicate experiments from Figure 2. See Figure 2 legend.

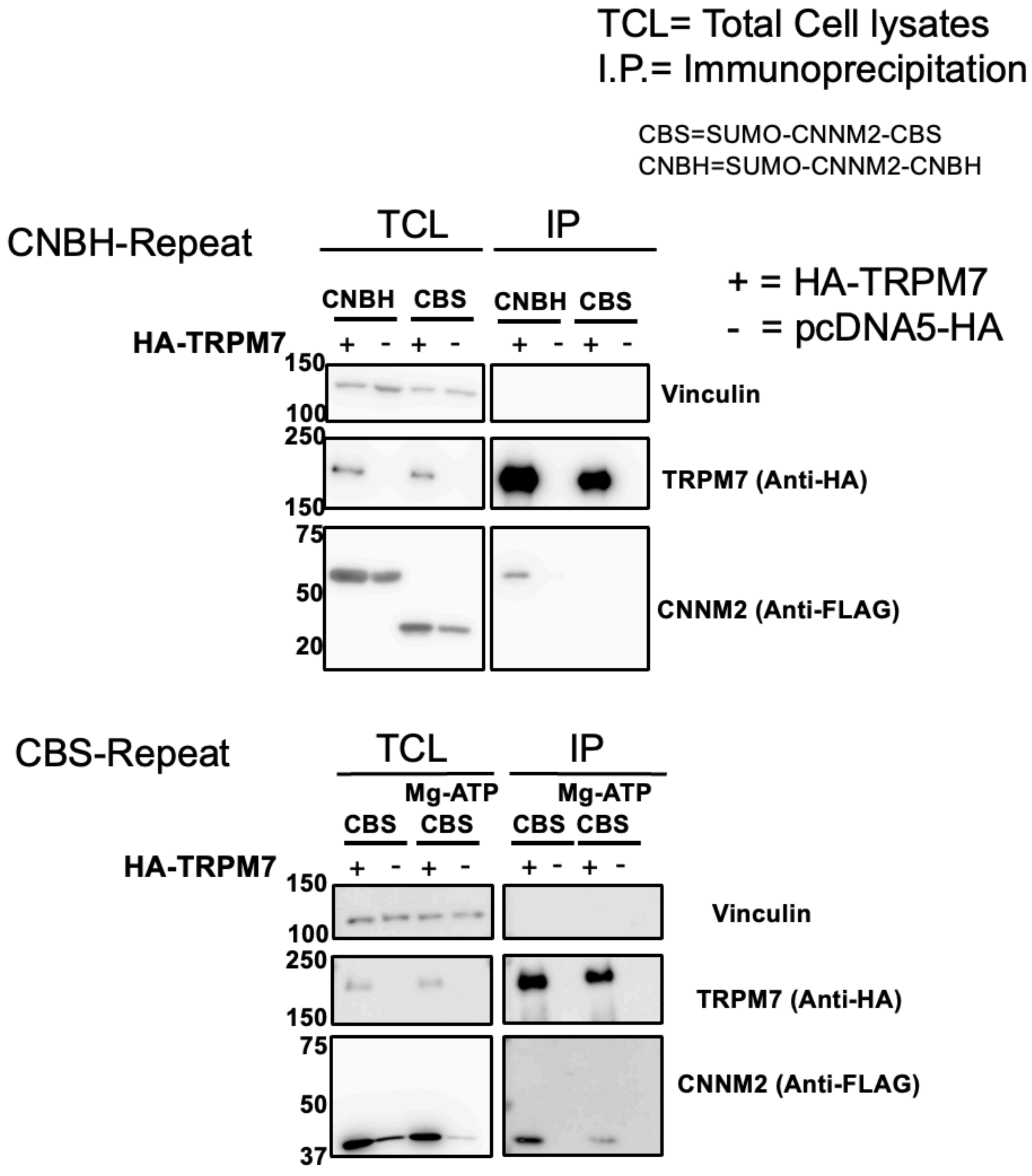

Figure 2 Replicate

Figure S6 – Replicate experiments from Figure 3. See Figure 3 legend.

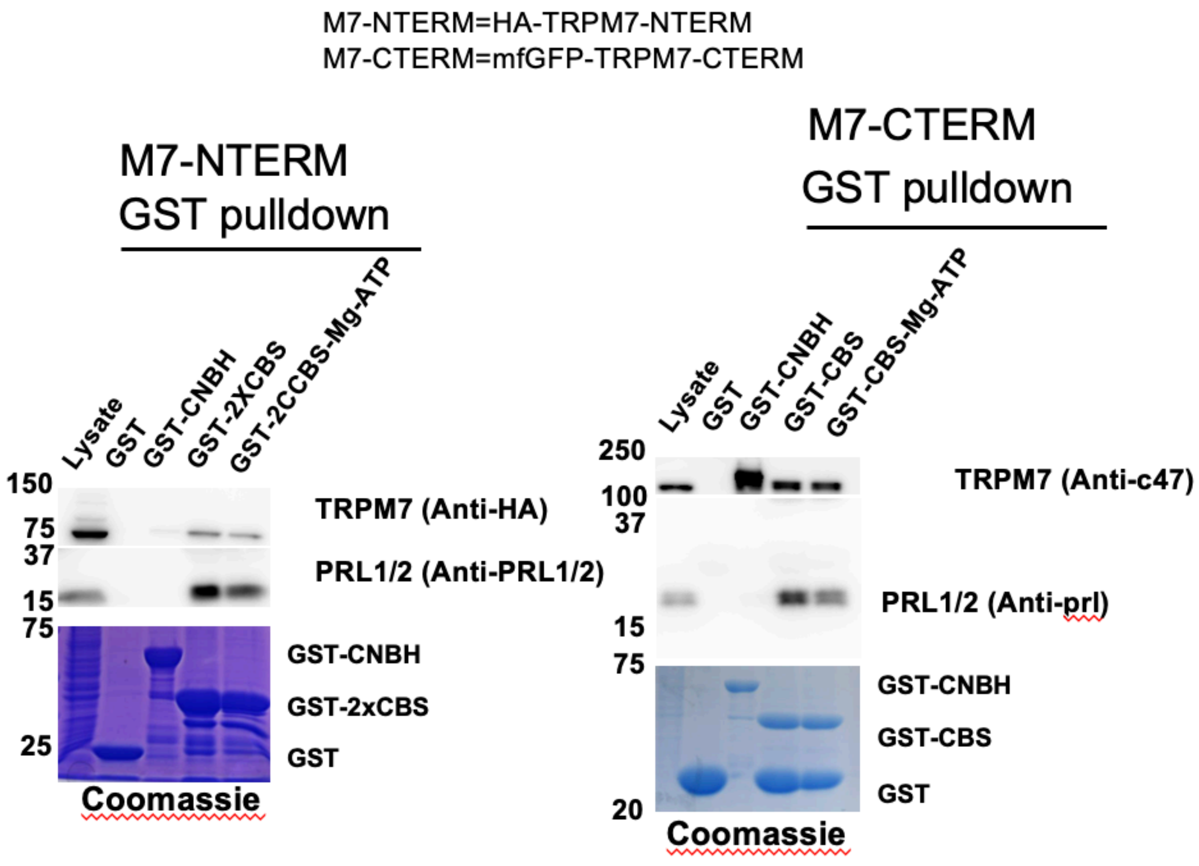

Figure 3B Replicate

Figure S6– Replicate experiments from Figure 3. See Figure 3 legend.

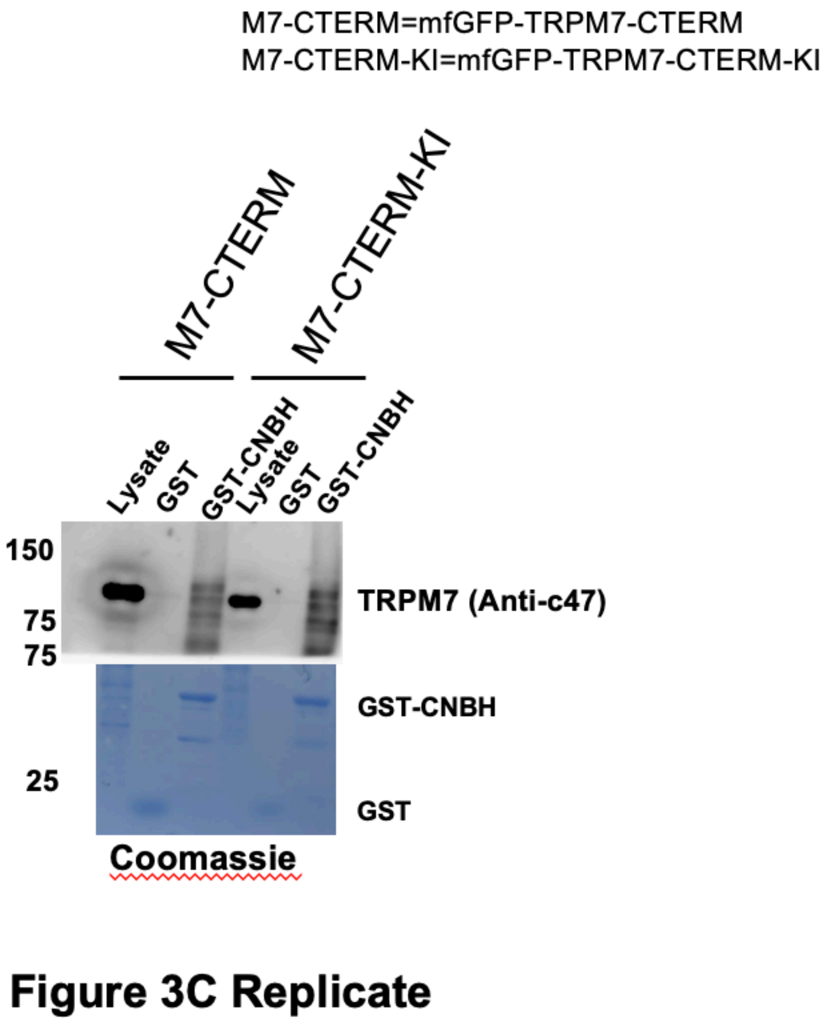

Figure S6 – Replicate experiments from Figure 3. See Figure 3 legend.

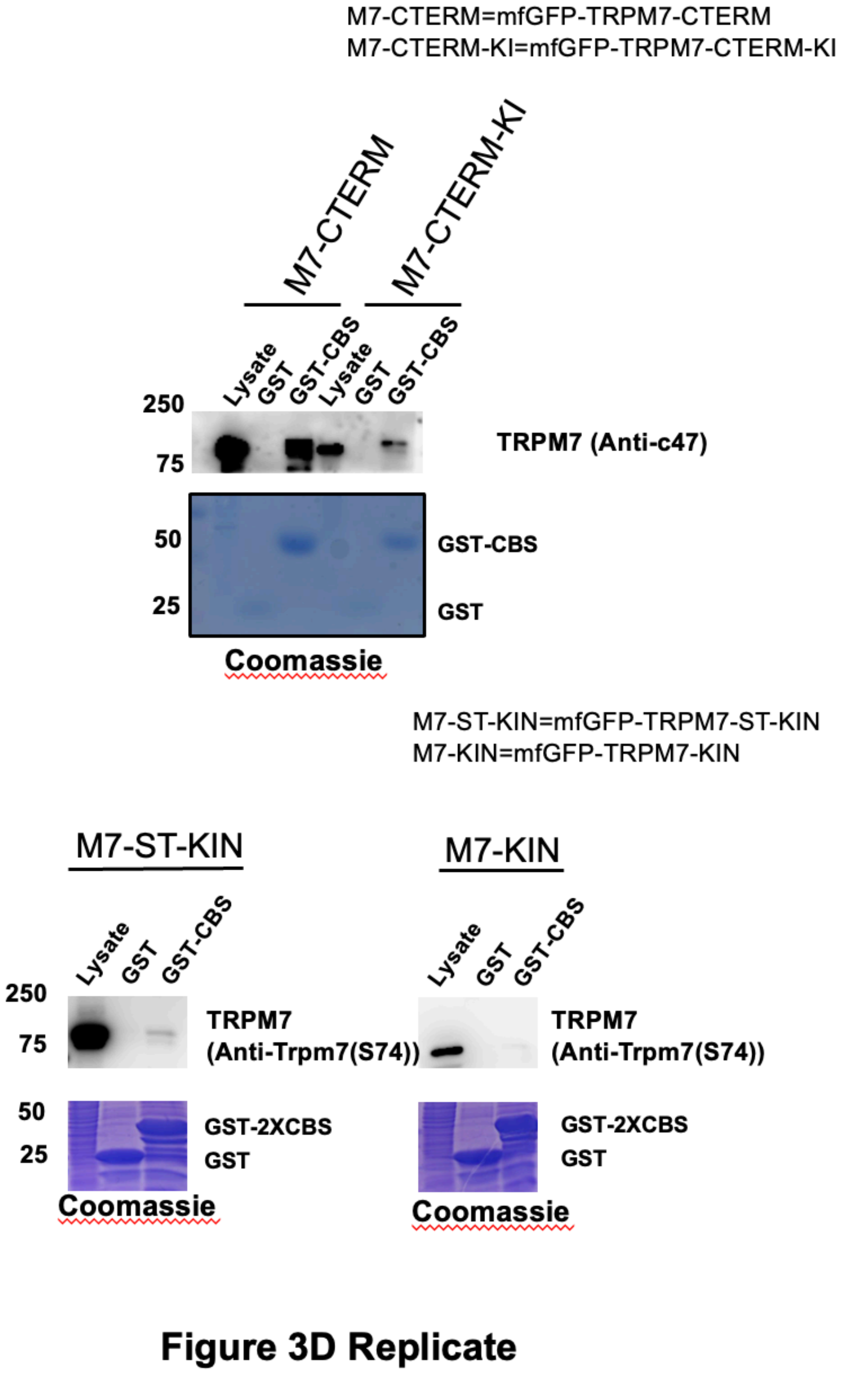

Figure S6 – Replicate experiments from Figure 3. See Figure 3 legend.

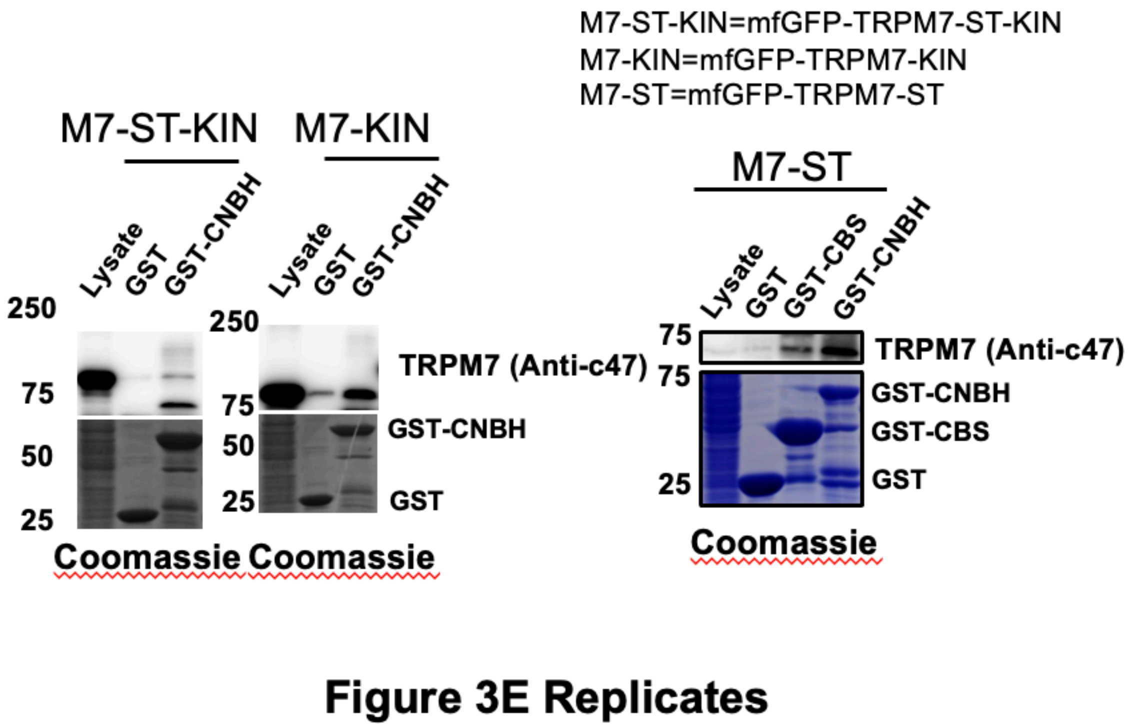

Figure S7. CNNM2's CNBH domain reproducibly stimulates TRPM7 kinase activity.

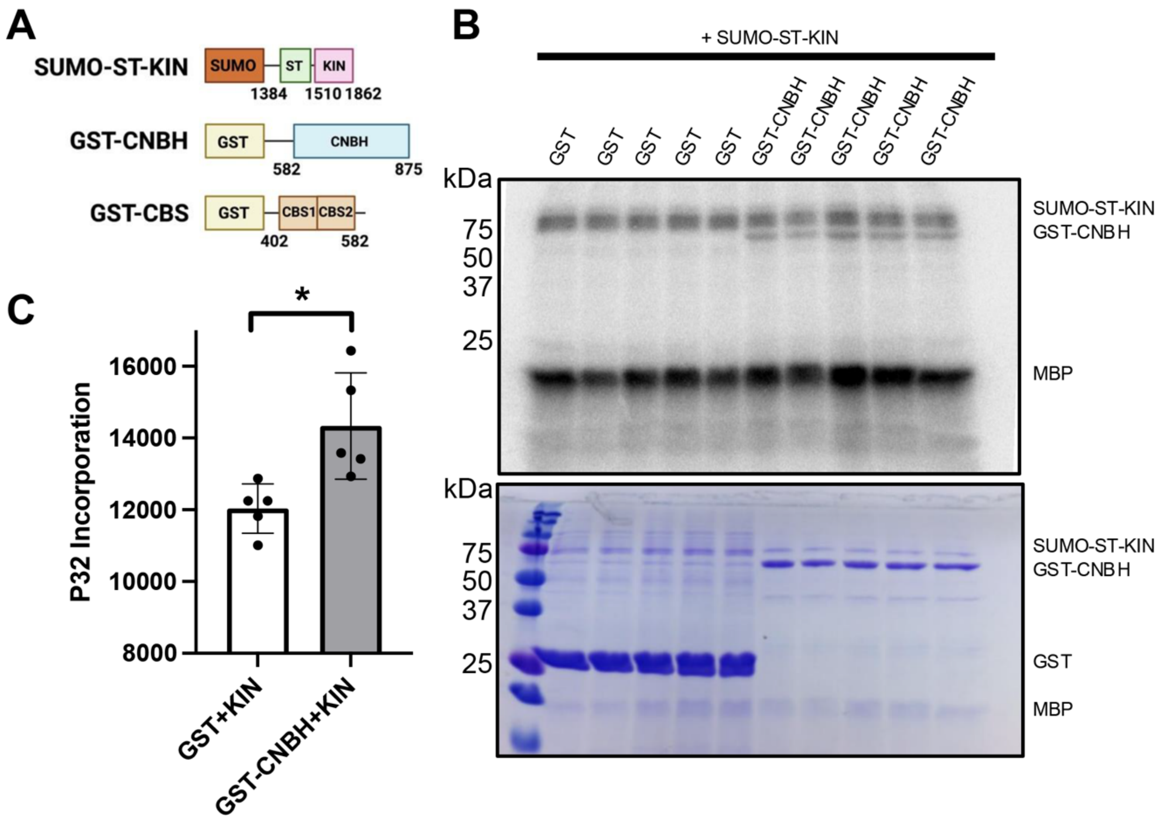

Figure S7. CNNM2's CNBH domain reproducibly stimulates TRPM7 kinase activity.

(A) Schematics of protein constructs used in the following *in vitro* kinase assay. **(B)** Bacterially purified SUMO-tagged TRPM7 fragment containing portions of the ST-region and catalytic kinase domain (SUMO-ST-KIN) was used in an *in vitro* kinase assay using myelin basic protein (MBP) as a generic substrate. The GST-tagged CNBH (GST-CNBH) domain was included to assess its effect on the catalytic activity of the TRPM7 kinase. GST alone was used as a negative control. The autoradiograph shows incorporation of ^{32}P into MBP, SUMO-M7-ST-KIN, and GST-CNBH. Phosphorylation of GST-CNBH, but not GST, indicate that the CNBH domain is a substrate of the TRPM7 kinase. **A** Coomassie-stained SDS-PAGE gel shows proteins included in the *in vitro* kinase assay after a 6-minute reaction. **(C)** Quantification of the ^{32}P incorporation into MBP from (B). GST-CNBH modestly stimulated the kinase activity of SUMO-ST-KIN (p=0.0245, unpaired two-tailed t-test). The statistical analysis was performed using GraphPad Prism version 10.0.1 (218) with * indicating $p \leq 0.05$.
